# Ryanodine Receptor Activation Induces Long-Term Plasticity of Spine Calcium Dynamics

**DOI:** 10.1371/journal.pbio.1002181

**Published:** 2015-06-22

**Authors:** Friedrich W. Johenning, Anne-Kathrin Theis, Ulrike Pannasch, Martin Rückl, Sten Rüdiger, Dietmar Schmitz

**Affiliations:** 1 Neuroscience Research Center, Charité-Universitätsmedizin, Berlin, Germany; 2 Berlin Institute of Health (BIH), Berlin, Germany; 3 Institute of Physics, Humboldt Universität, Berlin, Germany; 4 Bernstein Center for Computational Neuroscience, Berlin, Germany; 5 Cluster of Excellence ‘NeuroCure’, Charité-Universitätsmedizin, Berlin, Germany; 6 DZNE- German Center for Neurodegenerative Diseases, Berlin, Germany; The Salk Institute for Biological Studies, UNITED STATES

## Abstract

A key feature of signalling in dendritic spines is the synapse-specific transduction of short electrical signals into biochemical responses. Ca^2+^ is a major upstream effector in this transduction cascade, serving both as a depolarising electrical charge carrier at the membrane and an intracellular second messenger. Upon action potential firing, the majority of spines are subject to global back-propagating action potential (bAP) Ca^2+^ transients. These transients translate neuronal suprathreshold activation into intracellular biochemical events. Using a combination of electrophysiology, two-photon Ca^2+^ imaging, and modelling, we demonstrate that bAPs are electrochemically coupled to Ca^2+^ release from intracellular stores via ryanodine receptors (RyRs). We describe a new function mediated by spine RyRs: the activity-dependent long-term enhancement of the bAP-Ca^2+^ transient. Spines regulate bAP Ca^2+^ influx independent of each other, as bAP-Ca^2+^ transient enhancement is compartmentalized and independent of the dendritic Ca^2+^ transient. Furthermore, this functional state change depends exclusively on bAPs travelling antidromically into dendrites and spines. Induction, but not expression, of bAP-Ca^2+^ transient enhancement is a spine-specific function of the RyR. We demonstrate that RyRs can form specific Ca^2+^ signalling nanodomains within single spines. Functionally, RyR mediated Ca^2+^ release in these nanodomains induces a new form of Ca^2+^ transient plasticity that constitutes a spine specific storage mechanism of neuronal suprathreshold activity patterns.

## Abstract 2

A combination of two-photon calcium imaging, electrophysiology, and modelling shows how ryanodine receptors (a type of intracellular calcium channel) generate a signalling nanodomain within individual dendritic spines, enabling compartmentalized plasticity of calcium dynamics.

## Author Summary

Experiences change neuronal circuits, and these circuit changes outlast the initial experiences. This means that, in neurons, the fast electrical activity encoding experiences needs to be transduced into longer-lived biochemical and structural changes. A key mediator between these two timescales of neuronal activity is the Ca^2+^ ion. Ca^2+^ serves both as an electric charge carrier mediating fast voltage changes at the membrane and as a second messenger activating intracellular signalling cascades. Even within the spatial confines of dendritic spines, the specialized domains of dendrites that receive synaptic connections, Ca^2+^ encodes a versatile array of specific functions. In this study, we first demonstrate that voltage-gated Ca^2+^ channels and ryanodine receptors, intracellular channels located on the membrane of the endoplasmic reticulum through which Ca^2+^ can be released into the cytosol, are electrochemically coupled in single dendritic spines. We identify how ryanodine receptors induce enhancement of the Ca^2+^ influx, mediated by the opening of voltage-gated Ca^2+^ channels, induced by action potentials in a compartmentalized, spine-specific manner. Within the femtoliter volume of a single spine, specificity of this route of Ca^2+^-signalling is achieved by a signalling nanodomain centred on the ryanodine receptor. Our work stresses the role of the ryanodine receptor not only as an ion channel releasing Ca^2+^ from the endoplasmic reticulum but also as a macromolecular complex generating specificity of Ca^2+^-signalling within the spatial constraints of a single spine.

## Introduction

A fundamental principle in neuronal signal processing is the transduction of short electrical signals at the membrane into biochemical responses, resulting in longer lasting changes of the neuronal structural and functional state. Ca^2+^ molecules have a key role in this transfer process; they transduce electrical signals at the membrane into second messenger pathways involved in a number of adaptive responses. These include changes in synaptic strength, membrane excitability, cell morphology, and gene expression [1]. Versatility and specificity of this promiscuous messenger is achieved by differential signalling in space, time, and amplitude [2].

Ca^2+^ mediated transduction processes have a major impact on structure–function relationships in dendritic spines, which host the majority of the excitatory postsynaptic machinery [3]. Spines serve as independent biochemical and electrical functional units that provide synapse specific Ca^2+^ signals [4]. This compartmentalization results in the transduction of neuronal activity into a spine-specific Ca^2+^ code. Changes of this spine Ca^2+^ code constitute a change of a spine's functional state. It is therefore important to understand the regulation and dynamics of spine Ca^2+^ signalling.

Spine Ca^2+^ influx via the plasma membrane is mediated by N-methyl-D-aspartate receptors (NMDARs) and voltage gated Ca^2+^ channels (VGCCs). Suprathreshold neuronal activity, resulting in action potential (AP) firing, produces two different activation modes for spine Ca^2+^ influx. A subset of spines receives local synaptic input evoked by excitatory synaptic transmission, resulting in NMDAR- and VGCC- mediated Ca^2+^ influx [5]. Per given suprathreshold activation sequence, the majority of spines, however, do not receive specific synaptic activation. In these spines, depolarization mediated by back-propagating APs (bAPs) activates VGCCs. This signalling mode is independent of presynaptic input and affects a large population of spines throughout the dendritic tree [6]. To date, this unspecific, bAP-mediated signalling mode is poorly understood.

In addition to Ca^2+^ influx from the outside via voltage- and ligand-gated ion channels, specific synaptic activation can cause the recruitment of intracellular Ca^2+^ stores. This results in Ca^2+^ release from the endoplasmic reticulum (ER) via intracellular Ca^2+^ release channels (the inositol-trisphosphate and/or ryanodine receptor [IP3R and RyR]) in spines [5,7–12] and dendrites [13]. Here, we identify the recruitment of intracellular Ca^2+^ release channels by bAP-mediated Ca^2+^ transients in spines of excitatory cortical neurons, which has, to our knowledge, not been demonstrated so far [1,9,14] (but see [15] for RyR contribution to bAPs in gabaergic striatal spiny neuron spines).

We studied the activity-dependent temporal dynamics of bAP-Ca^2+^ transients in spines that do not receive specific synaptic inputs but undergo unspecific activation by bAPs in excitatory cortical and hippocampal neurons. We describe a new compartmentalized plastic change of a spine's functional state: the suprathreshold activity-dependent enhancement of bAP-Ca^2+^ transients. The induction of enhancement in spines requires RyR mediated Ca^2+^ release that is organized in a functional nanodomain.

## Results

### Electrochemical Coupling between bAPs and Intracellular Ca^2+^ Release in Spines

**Fig 1:**
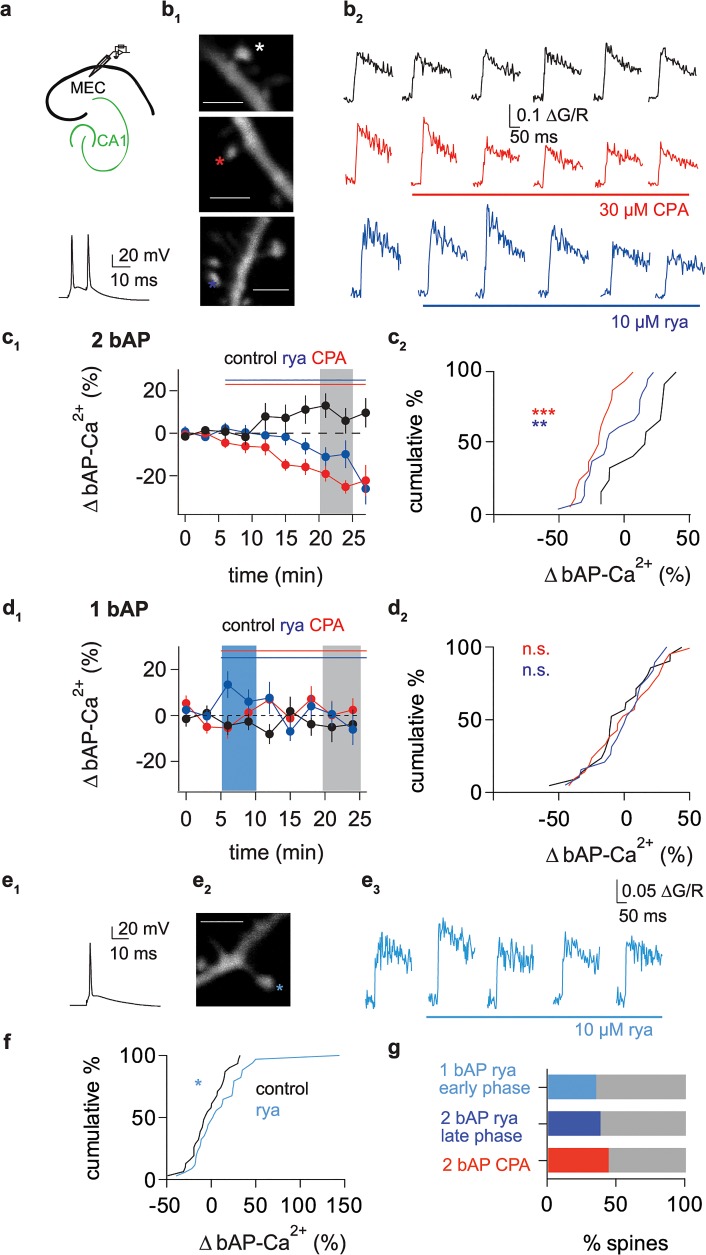
Electrochemical coupling of bAPs and intracellular Ca^2+^ store release in dendritic spines. (a) Top: Illustration of recording pipette positioning in layer 2 of the MEC. Bottom: Representative voltage trace of an AP doublet (100 Hz) evoked by current injection to induce the bAP-Ca^2+^ transients displayed in b_2_. (b_1_) Z-projection of the imaged dendritic segments in MEC layer 2 under control conditions (top), with CPA wash-in (middle), and with ryanodine wash-in (bottom; scale bars correspond to 2 μm). Asterisks mark imaged spines. (b_2_) Averaged traces (corresponding to consecutive 5 min recording intervals) of a control spine (black, top), a spine in the presence of 30 μM CPA as indicated by the red bar (red, middle), and a spine in the presence of 10 μM ryanodine (blue, bottom). (c_1_) Time plot of normalized doublet evoked bAP-Ca^2+^ amplitudes comparing control (black), CPA wash-in (red), and ryanodine wash-in (blue) after 5 min of baseline (indicated by the red bar). One doublet was applied every 60 s; the data is plotted in 3 min bins. Interval used for normalized post/pre ratios of bAP-Ca^2+^ is shaded in grey. (c_2_) Cumulative distribution plot of the averaged bAP-Ca^2+^ amplitudes 20 to 25 min after stimulation onset (corresponding to 16 to 21 min after drug wash-in; grey area in c_1_) normalized to baseline. Reduction by CPA (red, -21 ± 3%, *n* = 16/3 spines/cells) and ryanodine (blue, -10 ± 5%, *n* = 21/5 spines/cells) is significant when compared to the same time interval under control conditions (black, +11 ± 6%, *n* = 12/3 spines/cells, CPA versus control: *p* < 0.001, ryanodine versus control: *p* < 0.01, ANOVA with Bonferroni's Multiple Comparison Test). (d_1_) Time plot of normalized single AP-evoked bAP-Ca^2+^ amplitudes comparing control (black), CPA wash-in (red), and ryanodine wash-in (blue) after 5 min of baseline (indicated by red bar). One AP was applied every 60 s; the data is plotted in 3 min bins. Interval used for normalized post/pre ratios of bAP-Ca^2+^ in d_2_ is shaded in grey. (d_2_) Cumulative distribution plot of the averaged bAP-Ca^2+^ amplitudes 15 to 20 min after drug wash-in (grey area in d_1_) normalized to baseline. Reduction by CPA (red, *n* = 21/5 spines/cells) and ryanodine (blue, *n* = 19/6 spines/cells) is not significant when compared to the same time interval under control conditions (black, *n* = 21/5 spines/cells, not significant (n.s.), ANOVA with Bonferroni's Multiple Comparison Test). (e_1_) Representative voltage trace of a single AP evoked by current injection to induce the bAP-Ca^2+^ transient displayed in e_3_. (e_2_) Z-projection of the imaged dendritic segment (scale bar corresponds to 2 μm). Asterisk marks imaged spine. (e_3_) Averaged single AP-traces before and after wash-in of 10 μM ryanodine as indicated by the blue bar. (f) Cumulative distribution plot of the averaged single bAP-Ca^2+^ amplitudes in the first 5 min of drug wash-in (blue area in d_1_) normalized to baseline. The increase in the initial phase of ryanodine wash-in (+10 ± 6%, *n* = 34/8 spines/cells) is significant when compared to the same time interval under control conditions (-4 ± 4%, *n* = 31/7 spines/cells, *p* < 0.05, one-tailed Mann Whitney U test). (g) Bar graph of the fraction of spines responding with an effect size >1 standard deviation than the time-matched controls for 1 bAP during the first 5 min of ryanodine wash-in (35%, light blue), 2 bAPs 20 to 25 min after stimulation onset with ryanodine (38%, dark blue) and CPA (44%, red) wash-in. Data are expressed as mean standard error of the mean (SEM) * *p* < 0.05; ** *p* < 0.01; *** *p* < 0.001.

We performed two-photon Ca^2+^ imaging of bAP-Ca^2+^ transients in spines and adjacent dendritic segments. In acute brain slices, we studied cortical neurons in layer 2 of the medial entorhinal cortex (MEC) using fluo-5F (500 μM) as a Ca^2+^ indicator (Fig 1A). The contribution of RyR-mediated Ca^2+^ release to doublet bAP-Ca^2+^ transients was analysed using different pharmacological approaches (Fig 1B and 1E).

Cyclopiazonic acid (CPA) depletes intracellular Ca^2+^ stores by blocking the sarcoplasmic/endoplasmic reticulum calcium ATPase (SERCA) [16]. Doublet bAP-Ca^2+^ transients were evoked and imaged every 60 s. CPA significantly reduced bAP-Ca^2+^ transients 16 min after wash-in compared to time-matched controls (Fig 1C). As a SERCA blocker, CPA also interferes with intraspine Ca^2+^ clearance and significantly prolongs the decay time constant τ (S1 Fig). To directly block the RyR intracellular Ca^2+^ release channel, we applied 10 μM of ryanodine, a concentration that blocks RyRs [9,17,18]. After 16 min of ryanodine wash-in, bAP-Ca^2+^ transients were significantly reduced compared to controls (Fig 1C). Control values 20 to 25 min after stimulation onset were not significantly different from a theoretical mean of 0% change. In contrast, the reduction by ryanodine (*p* < 0.05) and by CPA (*p* < 0.0001) were significantly different (one sample *t* test).

There was no correlation between the intracellular Ca^2+^ release-related reduction of the bAP- Ca^2+^ transient and the apparent spine size (S1 Fig). This indicates intracellular Ca^2+^ stores in spines of different sizes. Reduction was, however, correlated with the initial amplitude of the bAP-Ca^2+^ transient. Thus, spines with large bAP-Ca^2+^ transients more likely demonstrate intracellular Ca^2+^ store release (S1 Fig). Ca^2+^ stores are an individual property of dendritic spines, as spines responding to CPA or ryanodine were found on the same dendritic segments as non-responders.

Store release via RyRs requires activation of the RyR by intracellular Ca^2+^ rises above a certain threshold [15,19]. We tested whether intracellular stores could also be activated when applying single APs instead of doublets. Single bAP-Ca^2+^ transients did not cross the threshold for RyR activation, as they were not significantly reduced by CPA or ryanodine (Fig 1D).

To test whether this finding is in line with the biophysical properties of the RyR, we modelled RyR open probability as a function of VGCC mediated Ca^2+^ influx during bAPs. The statistical model of single RyR opening corroborates our experimental finding. A 50% reduction of the doublet bAP-Ca^2+^ transient amplitude results in a 7-fold decrease in channel open probability (S1 Fig; see Methods for model parameters). Our experimental observation is confounded by the high buffer capacity introduced with fluo-5F. It is conceivable that the threshold for RyR activation is crossed by a single AP when the buffer is reduced. However, although initial RyR opening is an all-or-none phenomenon, the time window in which the channel is activated depends on the duration of the VGCC mediated stimulation transient. Therefore, doublets will still result in a longer time window of RyR activation, causing a larger RyR mediated Ca^2+^ response when considering the steady state open probability over time (S1 Fig).

At sub-μM concentrations, ryanodine has been reported to activate the RyR by locking it in a subconductance state [20]. In RyR type 2, this low ryanodine activation has been demonstrated to enhance the channel's sensitivity to [Ca^2+^] [21]. In the initial period after wash-in of 10 µM ryanodine, the concentration in our brain slice preparation will be significantly lower (most likely in the sub-μM range), resulting in RyR activation. Under subthreshold conditions where [Ca^2+^] does not activate the channel, low ryanodine should lead to activation of the RyR. Indeed, under low ryanodine conditions, single bAPs evoke significantly larger bAP-Ca^2+^ transient amplitudes when compared to control conditions (Fig 1D_1_, 1E and 1F).

We next calculated the fractions of spines responding to our different pharmacological interventions to estimate the percentage of spines containing intracellular Ca^2+^ stores. A spine was classified as responsive when the effect size of the drug was larger than one standard deviation of the corresponding control values (21% decrease in the doublet bAP control group 20 to 25 min after the onset of stimulation, 20% increase in the 1 bAP control group for the first 5 min after baseline). Based on this criterion, Ca^2+^ release from RyRs contributed to bAP-Ca^2+^ transients in a large fraction of spines, indicative of the presence of intracellular Ca^2+^ stores in these spines (35%–44%, Fig 1G).

### bAP-Ca^2+^ Transient Plasticity in MEC Layer 2 and Hippocampal CA1 Spines

We observed a small trend towards bAP-Ca^2+^ transient run-up at the end of our control timelines, which is, however, not significantly different from a theoretical mean of 0% change when using a one sample *t* test (Fig 1C_1_). We next wanted to design an experimental protocol that permitted us to evoke significant bAP-Ca^2+^ transient enhancement. For these experiments, we switched to the low affinity calcium indicator fluo-4FF. We reasoned that a low concentration (200 μM) of an indicator with a K_d_ of 10 μM would result in a low enough buffer capacity to be able to observe downstream biological effects of the measured Ca^2+^ transients.

**Fig 2:**
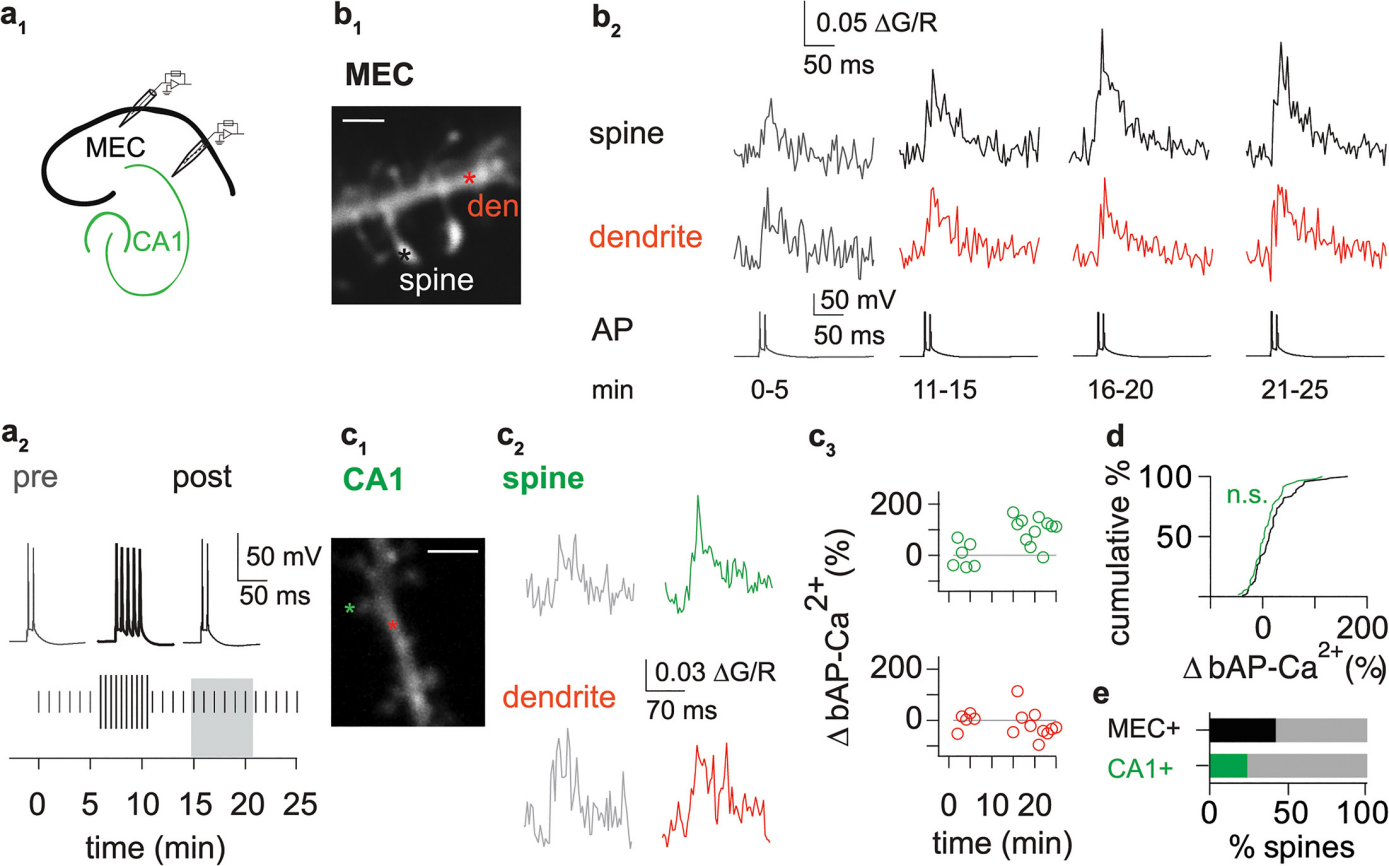
Enhancement of bAP-Ca^2+^ transients in dendritic spines. (a_1_) Illustration of recording pipette positioning in layer 2 of the MEC (black) and in the hippocampal CA1 region (green). (a_2_) Experimental paradigm for enhancement of bAP-Ca^2+^ transients. Top: Test stimulus AP doublets evoked by current injection before (pre, grey) and after (post, black) 5 AP bursts (bold black). Bottom: Experimental timeline, vertical bars correspond to test stimulus AP doublet (short bars) and 5 APs (long bars). Doublet test stimuli were delivered every 60 s. After a 6 min baseline measurement, we applied a bursting paradigm consisting of 5 APs delivered every 30 s for 5 min. After the bursting paradigm, we again measured the bAP-Ca^2+^ transient evoked by the doublet test stimulus. Grey box illustrates the interval 15 to 20 min after bAP stimulation onset which is compared to baseline stimulation to quantify enhancement. (b_1_) Z-projection of the imaged dendritic segment (scale bar corresponds to 2 μm). Asterisks mark imaged spine and dendritic segment. (b_2_) bAP Ca^2+^ transient enhancement in a cortical neuron. Averaged fluorescence traces of Ca^2+^ transients in spine (top) and dendrite (middle). Representative AP traces from the time intervals averaged for Ca^2+^ imaging (bottom). (c_1_) Z-projection of the imaged dendritic segment in a hippocampal CA1 pyramidal cell. (scale bar corresponds to 2 μm). Asterisks mark imaged spine and dendritic segment. (c_2_) Overlay of averaged fluorescence traces 0–5 (grey) and 15–20 min after bAP stimulation onset in a CA1 pyramidal cell spine (green) and the adjacent dendrite (red). (c_3_) Time plot of normalized single sweep amplitudes. (d) Cumulative distribution plot of normalized bAP Ca^2+^ transient enhancement in spines of MEC layer 2 cortical neurons (19 ± 4%, *n* = 92/43 spines/cells, black) and hippocampal CA1 pyramidal cells (8 ± 4%, *n* = 59/22 spines/cells, green), comparison did not reach significance (n.s., Mann Whitney U Test). (e) Bar graph illustrates percentage of spines displaying bAP-Ca^2+^ transient enhancement >20% in MEC layer 2 cells (42%, 39/27 out of 92/43 spines/cells, black) and in hippocampal CA1 pyramidal cells (24%, 14/8 out of 59/22 measured spines/cells, green).

**Fig 3:**
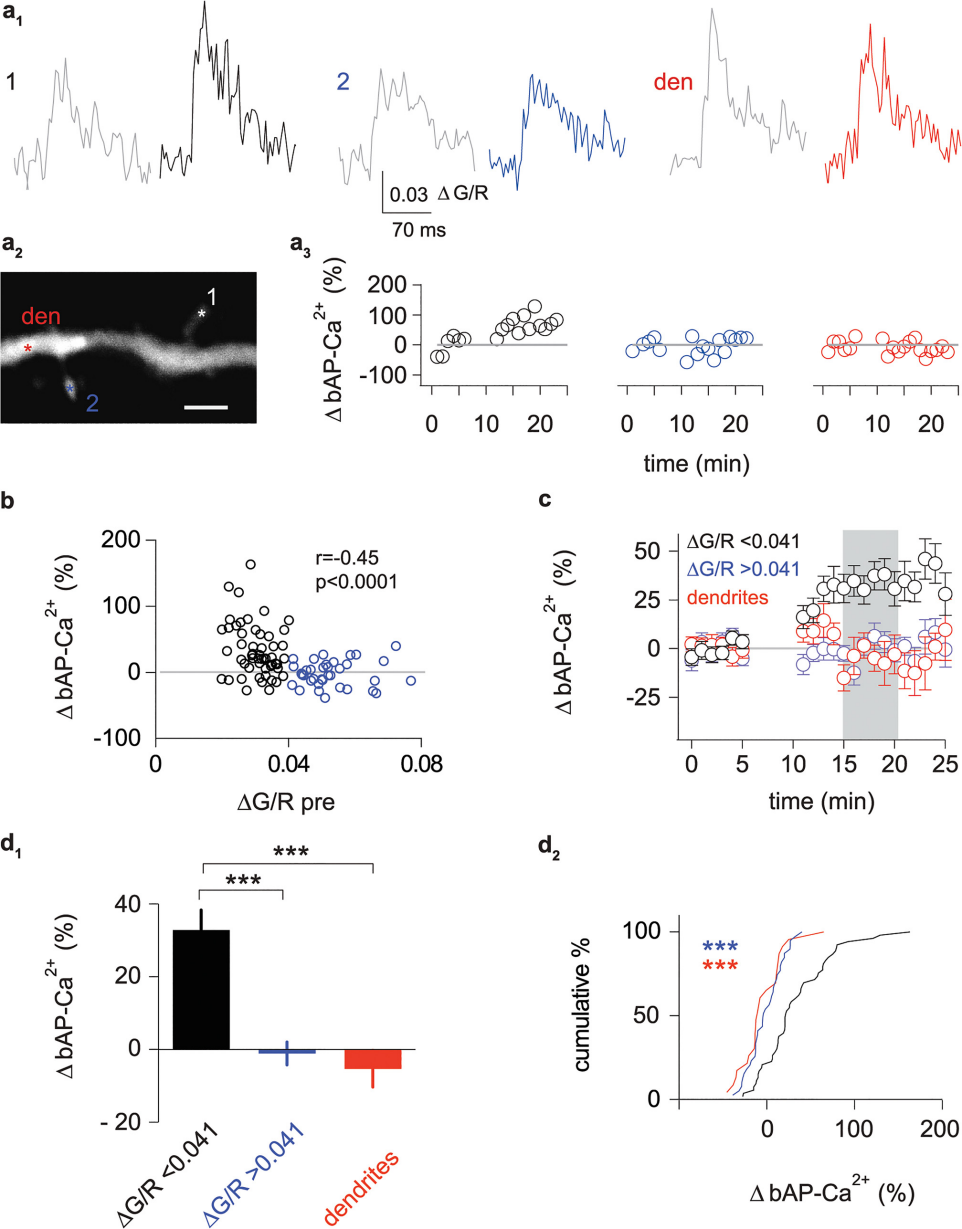
A subpopulation of spines undergoes activity-dependent enhancement. (a_1_) Averaged baseline bAP-Ca^2+^ transients 0 to 5 min after bAP stimulation onset (grey traces) and averaged bAP-Ca^2+^ transients 15 to 20 min after bAP stimulation onset of 2 adjacent spines (black and blue) and the dendrite (red). (a_2_) Z-projection of the imaged dendritic segment (scale bar corresponds to 2 μm). Asterisks mark imaged spines and dendritic segment. (a_3_) Time plots of normalized single sweep amplitudes. (b) The averaged baseline amplitude is plotted against bAP-Ca^2+^ transient enhancement 15 to 20 min after bAP stimulation onset (r = -0.45, *n* = 92/43 spines/cells, *p* < 0.0001, Spearman’s rank order test). Spines with pre induction amplitudes <0.041 ∆G/R are depicted in black (*n* = 53/31 spines/cells), spines with pre-induction amplitudes >0.041 in blue (*n* = 39/22 spines/cells). (c) Time plot of normalized bAP-Ca^2+^ transients demonstrates selective enhancement of spines with baseline amplitudes <0.041 ∆G/R (black) when compared to dendrites (red) and spines with baseline amplitudes >0.041 ∆G/R (blue). Interval 15 to 20 min after bAP stimulation onset used for quantification of normalized enhancement is shaded in grey. (d_1_) Bar graph of normalized enhancement 15 to 20 min after bAP stimulation onset. Enhancement for spines with baseline ∆G/R<0.041 (black, +33 ± 6%, *n* = 53/31 spines/cells) is significantly larger than in spines with baseline ∆G/R>0.041 (blue, -1 ± 3%, *n* = 39/22 spines/cells, *p* < 0.001) and dendrites adjacent to spines with baseline ∆G/R<0.041 (red, -5 ± 5%, *n* = 23/23, dendrites/cells, *p* < 0.001, Kruskal Wallis Test with Dunn’s posthoc comparison). (d_2_) Cumulative distribution plot of normalized enhancement 15 to 20 min after bAP stimulation onset (shaded grey in c). Data are expressed as mean SEM *** *p* < 0.001.

To quantify spine-specific enhancement, bAP-Ca^2+^ transient amplitudes following a 15 min stimulation paradigm (Fig 2A_2_) were normalized to the baseline amplitudes (Fig 3C; see Methods and S6 Fig on amplitude measurements). In layer 2 cells of the MEC, a population of 92 spines from 43 cells fulfilled our quality criteria for long-term measurements (see Methods). As opposed to our fluo-5F control doublet timelines from Fig 1C, enhancement under these conditions was significantly different from a theoretical median of 0% change in the time interval 15 to 20 min after the onset of stimulation (*p* < 0.0001, Wilcoxon-signed rank test). This new protocol enabled us to study enhancement in a more systematic fashion with a larger effect size in a shorter experimental time window. 42% of the spines could be classified as plastic, which means they displayed bAP-Ca^2+^ transient enhancement by >20%. Spines with changes of the bAP-Ca^2+^ transient of <20% were defined as static spines.

To see whether the observed bAP-Ca^2+^ transient enhancement is a more generalized phenomenon, we also tested hippocampal CA1 pyramidal cells (Fig 2C). Baseline bAP-Ca^2+^ transients were significantly smaller in CA1 pyramidal cells than in layer 2 cells of the MEC (S2 Fig). Overall enhancement of the layer 2 MEC spine population was not significantly different from the CA1 cell spines (Fig 2D). However, we observed enhancement of >20% in only 24% of hippocampal CA1 spines (Fig 2E). Based on the larger fraction of plastic spines in layer 2 of the MEC, we focused on these cells for a further mechanistic analysis.

### bAP-Ca^2+^ Transient Enhancement Is Compartmentalized and Spine-Specific

Only a subpopulation of spines displayed bAP-Ca^2+^ transient enhancement. Does this subpopulation of plastic spines form clusters on a dendritic segment? We performed simultaneous fast imaging of several spines using the multiple line scan method [22]. Seventy-eight spines from a total number of 29 dendritic segments were imaged simultaneously with at least one neighbouring spine (average number of 2.7 ± 0.1 spines/segment). Twenty-two of these segments harboured spines with enhancement of >20% (average number of 2.8 ± 0.2 spines/segment). Twelve out of 22 segments only displayed enhancement in 1 spine (average number of 2.9 ± 0.2 spines/segment). In 8 out of 22 segments we observed enhancement in 2 spines (average number of 2.6 ± 0.3 spines/segment). Concurrent enhancement of 3 spines was found in 2 out of 22 segments; the spine number in both segments was 3. In sum, plastic and static spines are neighbours on the same dendritic segment (Fig 3A), activity-dependent enhancement of bAP-Ca^2+^ transients is a compartmentalized property of individual spines.

### Properties of Spines Predisposed for bAP-Ca^2+^ Transient Enhancement

Our next aim was to identify spine-specific factors that determine whether a spine is plastic or static. We searched for correlations between the degree of bAP-Ca^2+^ transient enhancement and a number of independent parameters. There was neither a significant correlation between enhancement and spine-soma distance (S3 Fig), nor the apparent spine size (S3 Fig), nor the distance of the spine head from the parent dendrite (S3 Fig).

However, the baseline bAP-Ca^2+^ transient amplitude of a spine displayed a significant inverse correlation to the level of enhancement (Fig 3B). We used the baseline bAP-Ca^2+^ transient amplitude to define a subpopulation of plastic spines with a high probability of enhancement for further analysis. For grouping, spines with small baseline bAP-Ca^2+^ transients (<0.041 ∆G/R; *n* = 53/31 spines/cells) were separated from spines with large baseline transients (>0.041 ∆G/R; *n* = 39/22 spines/cells). The value 0.041 ∆G/R was chosen as a separation criterion since it reflects the median baseline bAP-Ca^2+^ transient amplitude of all spines for which a sequence of baseline doublets was obtained under drug-free conditions (S2 Fig). When comparing enhancement between spines with small and large baseline bAP-Ca^2+^ transients, only the subpopulation of small baseline spines displayed significant enhancement (Fig 3D). Similar relationships between baseline amplitude and enhancement could be observed in hippocampal CA1 pyramidal cells (S3 Fig).

In contrast to spines with small bAP-Ca^2+^ transients, adjacent dendrites were not significantly enhanced (Fig 3D). However, when using multiple line scans, the imaged dendritic segment can be at an appreciable distance from the spines of interest (see Figs 2B and 3A). This experimental design could miss enhancement of bAP-Ca^2+^ transients in dendritic microdomains adjacent (±1 μm) to enhanced spines. In a subpopulation of 21 spines, we measured spines and their adjacent dendritic microdomain. In this dataset, spines are significantly enhanced, but there is no correlation between enhancement in spines and dendrites (S3 Fig).

We infer that enhancement predominantly occurs in a subpopulation of spines defined by small baseline bAP-Ca^2+^ transients. Saturation of the Ca^2+^ indicator could be a confounding factor underlying the absence of enhancement in large amplitude spines. In this context, it is important that 95% of baseline ∆G/R values measured with fluo-4FF were smaller than 0.07, whereas the Gmax/R value under saturating [Ca^2+^] was at 0.89 ± 0.03 (see Methods for details). Unless otherwise noted, statistical comparisons of enhancement were henceforth performed between grouped spine populations with small baseline doublet bAP-Ca^2+^ transient amplitudes.

### bAP-Ca^2+^ Transient Enhancement Can Be Evoked by Naturally Occurring Spike Patterns and Depends on Neuronal Output

**Fig 4:**
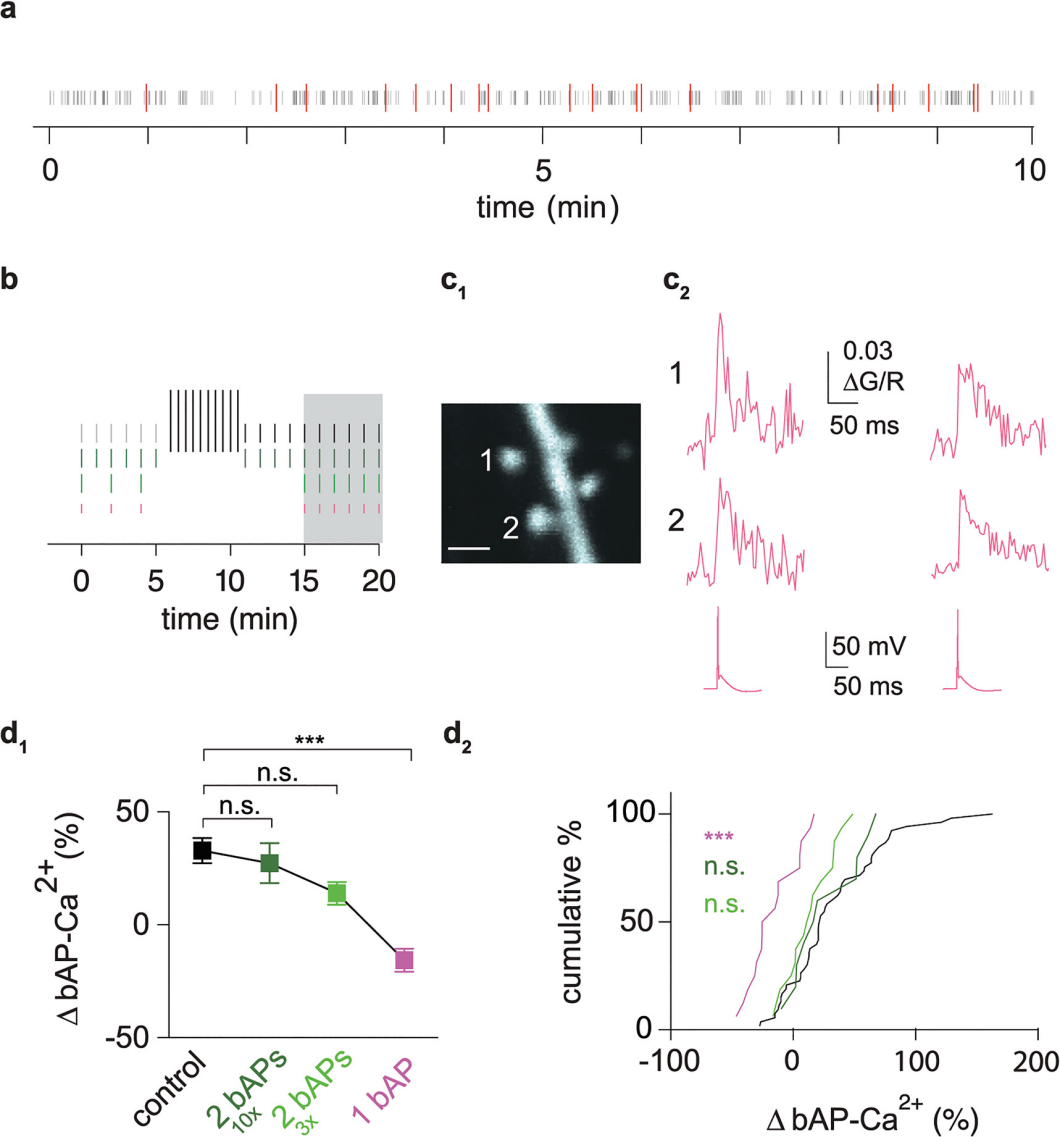
bAP-Ca^2+^ transient enhancement depends on neuronal output. (a) Raster plot of a 10 min in vivo extracellular recording from a freely moving p19 rat in layer 2 of the MEC. Grey bars correspond to single spikes. AP doublets with frequencies >100 Hz are indicated in red. (b) Diagram of experimental timelines. Grey and black vertical bars correspond to test stimulus doublets (short bars) and the ten 5 AP bursts administered after baseline (long bars). Dark green vertical bars depict doublet test stimuli delivered every 60 s. A 6 min baseline measurement was followed by a stimulus-free interval of 5 min. We then again measured the bAP-Ca^2+^ transients evoked by the doublet test stimulus. Light green bars correspond to three doublet test stimuli delivered at an interval of 120s and followed by a 10 min stimulus-free interval. After that, six doublets were measured at a 60 s interval. Shorter pink horizontal bars correspond to singlets. For baseline measurements, three singlet test stimuli were delivered at an interval of 120 s and followed by a 10 min stimulus-free interval. After that, six singlets were measured at a 60 s interval. Interval used for normalized post/pre ratios of bAP-Ca^2+^ transient enhancement is shaded in grey. (c_1_) Z-projection of the imaged dendritic segment (scale bar corresponds to 1 μm). (c_2_) Singlet evoked averaged bAP-Ca^2+^ transients from two neighbouring spines (top and middle). Bottom trace refers to representative underlying APs. (d_1_) Plot of normalized bAP-Ca^2+^ transient enhancement 15 to 20 min after bAP stimulation onset in spines with baseline ∆G/R <0.041. In the singlet experiments, the doublet response was measured at 20 min to permit grouping for comparison with the other doublet responses. bAP-Ca^2+^ transient enhancement in the control group (black, *n* = 53/31 spines/cells) is significantly larger than in the spine group where singlets were applied (-16.5%, *n* = 16/11 spines/cells, magenta, *p* < 0.001, Kruskal Wallis Test with Dunn’s posthoc comparison). Reduced enhancement upon application of doublets when 5 AP bursts were omitted (27 ± 8%, *n* = 10/6 spines/cells, dark green) and doublet number further reduced (14 ± 5%, *n* = 16/12 spines cells, light green). Both conditions were not significantly different from controls (n.s., Kruskal Wallis Test with Dunn’s posthoc comparison). (d_2_) Cumulative distribution plot of normalized bAP-Ca^2+^ transient enhancement. Dataset corresponds to d_1_. Data are expressed as mean SEM *** P < 0.001.

Next, we wanted to see whether enhancement conforms to realistic neuronal firing patterns and scales with AP output. Bursts of 5 APs in the 100 Hz range were not observed in a 10 min in vivo spike train from an MEC layer 2 cell displaying grid cell firing in a freely moving p19 rat (Fig 4A). As the in vivo spike train contained several 100 Hz doublets (Fig 4A), we investigated whether more physiological doublet firing induces a significant enhancement (ten doublets in 15 min, Fig 4B). Indeed, we still observed enhancement, which was not significantly different from control conditions using additional 5 AP bursts (Fig 4D).

A further reduction of the induction stimulus to a minimum of three doublets in 15 min resulted in reduced, albeit insignificantly different, enhancement when comparing to the control group (Fig 4D). We conclude that bAP-Ca^2+^ transient enhancement is a sensitive phenomenon that can be readily evoked by neuronal output patterns occurring in vivo.

Next, we wanted to test if a further reduction in neuronal output could abolish enhancement and whether doublet firing is required. We reduced the putative induction stimulus to three single bAPs (Fig 4C). Single AP stimulation was then continued after a 10 min stimulus-free interval (Fig 4B). The further reduction in bAP number and frequency abolished enhancement (Fig 4D). We even observed a small depression, which was significant when tested against a theoretical median of 0% change (*p* < 0.01, Wilcoxon signed rank test). Consequently, bAP-Ca^2+^ transient enhancement scales with neuronal output. For a further mechanistic analysis of the phenomenon, we adhered to our saturating 15 min stimulation protocol consisting of doublets and 5 AP bursts in order to maximise the robustness of bAP-Ca^2+^ transient enhancement: Including the 5 AP bursts, enhancement of >20% was reached in about 60% of spines in the small baseline bAP-Ca^2+^ transient amplitude. When omitting the 5 AP bursts, enhancement >20% was only observed in 40% of spines.

### bAP-Ca^2+^ Transient Enhancement Requires bAP-Mediated Intraspine Ca^2+^ Rises

**Fig 5:**
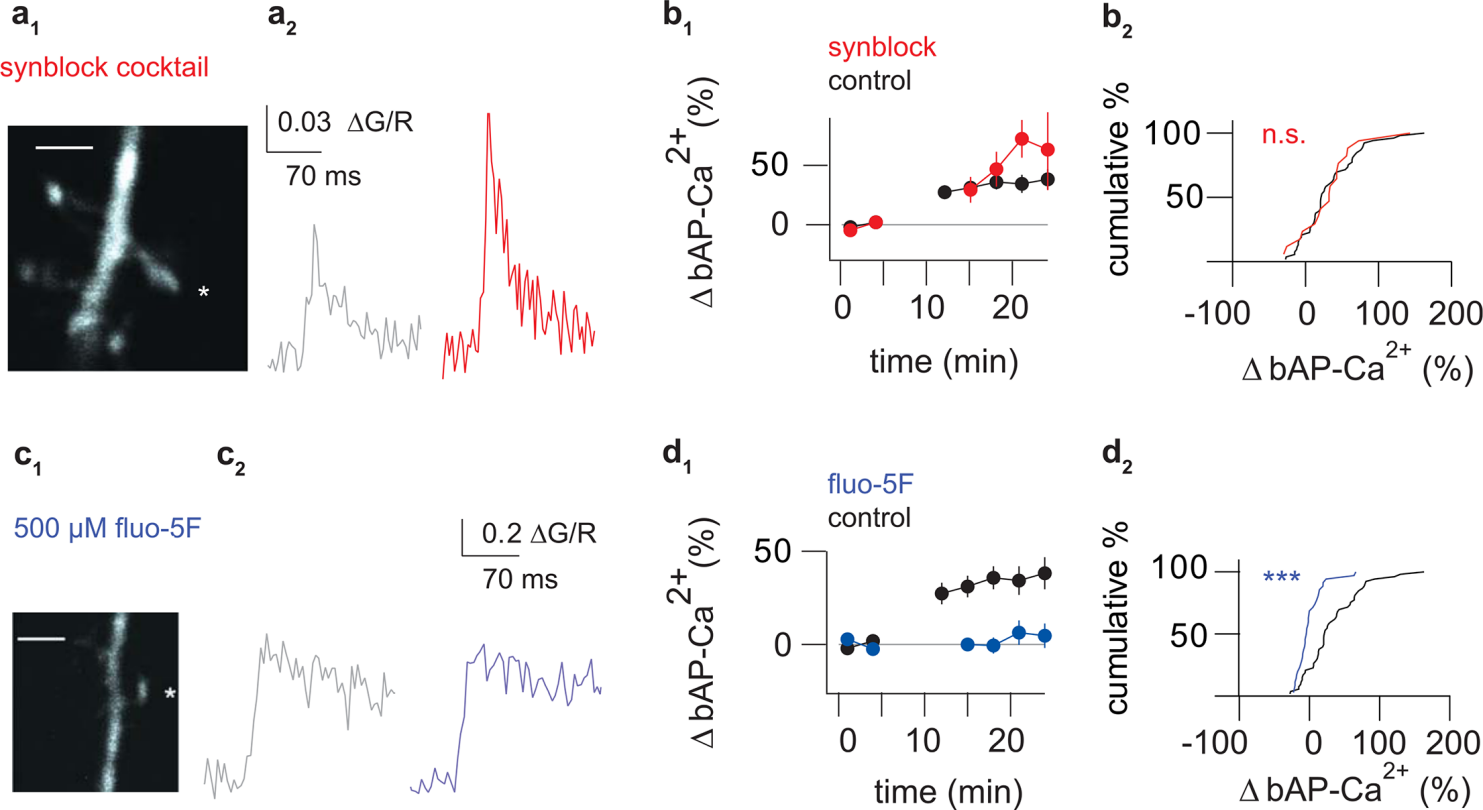
bAP-Ca^2+^ transient enhancement is independent of synaptic transmission and depends on bAP-mediated Ca^2+^ influx. (a_1_) Z-projection of the imaged spine (scale bar corresponds to 2 μm). Asterisk marks imaged spine. (a_2_) Averaged baseline bAP-Ca^2+^ transients 0 to 5 min after bAP stimulation onset (grey traces) and averaged bAP-Ca^2+^ transients 15 to 20 min after bAP stimulation onset (red) of a spine with synaptic transmission blocked using 1 μM Gabazine, 100 μM APV, 20 μM NBQX, and 500 μM (S)-MCPG/25μM MPEP and 100 μM LY 367385. (b_1_) Time plot of normalized doublet evoked bAP-Ca^2+^ transient enhancement in spines with small baseline amplitudes comparing control (black) spines to spines with synaptic transmission blocked (red). One doublet was applied every 60 s, the data is plotted in 3 min bins. In the synaptic transmission blocked group, doublets were applied 11 to 14 min after stimulation onset, but not imaged. (b_2_) Cumulative distribution plot of normalized bAP-Ca^2+^ transients enhancement 15 to 20 min after bAP stimulation onset in spines with small baseline amplitudes. Control spine enhancement (black, +33 ± 6%, *n* = 53/31 spines/cells) is not significantly different from spines with synaptic transmission blocked (red, +32 ± 10%, *n* = 17/9 spines/cells, *p* = 0.9, Mann Whitney U test). (c_1_) Z-projection of the imaged spine (scale bar corresponds to 2 μm). Asterisk marks imaged spine.(c_2_) Averaged baseline bAP-Ca^2+^ transients 0 to 5 min after bAP stimulation onset (grey traces) and averaged bAP-Ca^2+^ transients 15 to 20 min after bAP stimulation onset (blue) of a spine using 500 μM fluo-5F as a Ca^2+^ indicator. (d_1_) Time plot of normalized doublet evoked bAP-Ca^2+^ transient enhancement in spines comparing control spines measured with fluo-4FF (black) to spines measured with fluo-5F (blue). Fluo-5F data is selected based on the median baseline value of all spines with fluo-5F to match the fluo-4FF control group with small baseline bAP-Ca^2+^ transients. One doublet was applied every 60 s, the data is plotted in 3 min bins. In the fluo-5F group, doublets were applied 11 to 14 min after stimulation onset, but not imaged. (d_2_) Cumulative distribution plot of normalized bAP-Ca^2+^ transients enhancement 15 to 20 min after bAP stimulation onset in spines with small baseline amplitudes. Fluo-5F data is selected based on the median baseline value of all spines with fluo-5F to match the fluo-4FF control group with small baseline bAP-Ca^2+^ transients. Spines in the small pre-burst bAP-Ca^2+^ transient group also showed no enhancement with fluo-5F (blue, 1 ± 3%, *n* = 35/14 spines/cells) compared to the fluo-4FF control group (black, +33 ± 6%, *n* = 53/31 spines/cells; *p* < 0.001, Mann Whitney U Test). *** *p* < 0.001.

Induction and/or expression of bAP-Ca^2+^ transient enhancement could require pairing with spontaneous background synaptic transmission or tonic activation of γ-Aminobutyric acid type A (GABA-A) receptors, α-amino-3-hydroxy-5-methyl-4-isoxazolepropionic acid- (AMPA-) and NMDA receptors as well as metabotropic glutamate receptors (mGluRs). To address this possibility, we repeated our protocol with synaptic transmission blocked. No difference between the control group and the group with blocked synaptic transmission could be detected (Figs 5B and S4).

Most likely, bAP-mediated cytoplasmic Ca^2+^ elevations are the biochemical link between the induced membrane depolarization and downstream signal transduction events that result in enhancement. To test for an inductive role of free Ca^2+^, we again used 500 μM of the medium-affinity Ca^2+^ indicator fluo-5F, this time with an experimental protocol identical to the one that induces significant enhancement with fluo-4FF. At this concentration, free cytosolic Ca^2+^ is strongly buffered, permitting a linear readout of the spine Ca^2+^ flux while simultaneously reducing the activation of transduction cascades by intra-spine free Ca^2+^ (added buffer capacity k_dye_ of 350 for 500 μM fluo-5F according to Yasuda et al., 2004 [23]). Indeed, enhancement was blocked in the whole spine population measured with 500 μM fluo-5F (-10 ± 3%, *n* = 68/24 spines/cells; whole population 200 μM fluo-4FF: +19 ± 4%, *n* = 92/43 spines/cells; *p* < 0.001 Mann Whitney U Test).

We also grouped the fluo-5F data based on the median baseline bAP-Ca^2+^ transient value of all spines with fluo-5F (0.249 ∆G/R, *n* = 98/28 spines/cells) to match the fluo-4FF control group with small baseline values. Enhancement in the small bAP-Ca^2+^ transient group was also significantly smaller compared to the control group (Fig 5D).

Using 500 μM fluo-5F, the Gmax/R value under saturating [Ca^2+^] was at 1.34 ± 0.2 (see above and Methods for details). We infer that activity dependent enhancement requires sufficient free intraspine Ca^2+^.

### bAP-Ca^2+^ Transient Enhancement Depends on Activation of RyRs by bAPs

**Fig 6:**
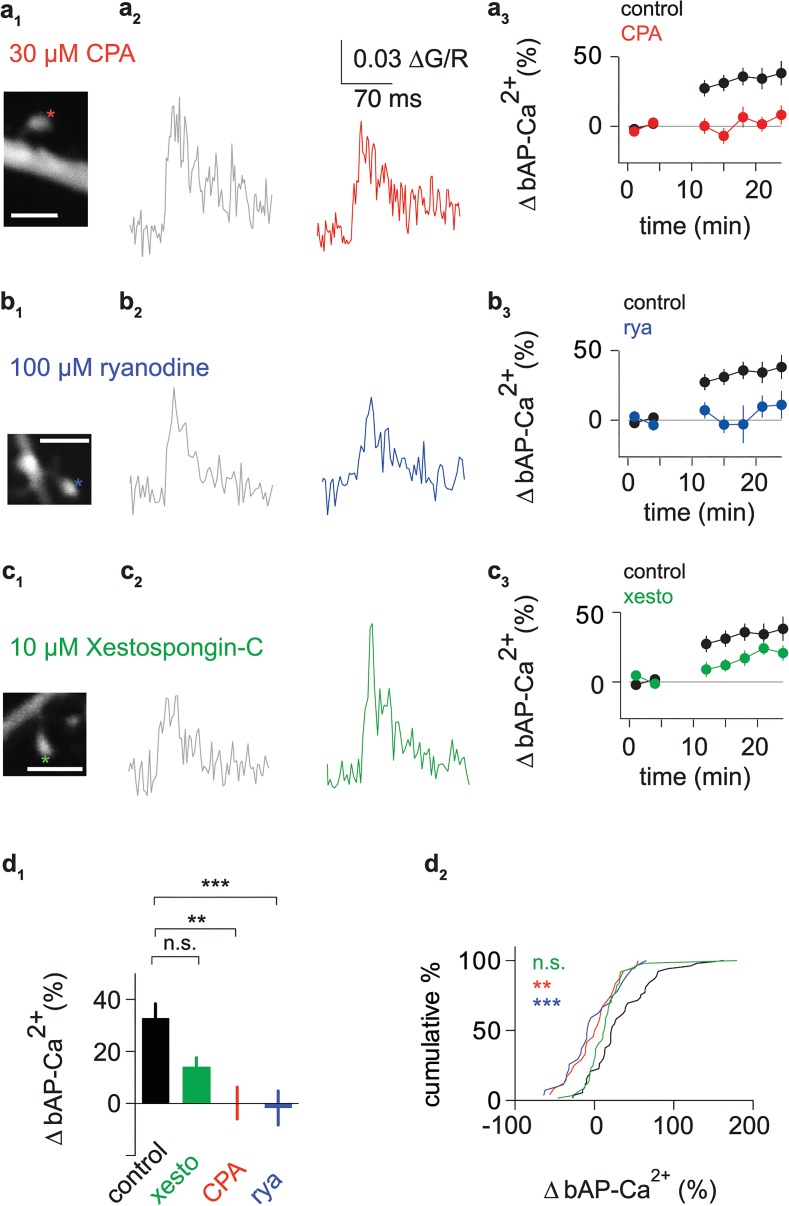
bAP-Ca^2+^ transient enhancement depends on Ca^2+^ release from intracellular stores. (a_1_) Z-projection of the imaged spine segment (scale bar corresponds to 2 μm). Asterisk marks imaged spine. (a_2_) Averaged baseline bAP-Ca^2+^ transients 0 to 5 min after bAP stimulation onset (grey) and averaged bAP-Ca^2+^ transients 15 to 20 min after bAP stimulation onset (red) of a spine in the presence of 30 μM CPA. (a_3_) Time plot of normalized doublet evoked bAP-Ca^2+^ transient enhancement in spines with small baseline amplitudes comparing control (black) spines to spines in the presence of 30 μM CPA (red). One doublet was applied every 60 s, the data is plotted in 3 min bins. (b_1_) Z-projection of the imaged spine segment (scale bar corresponds to 2 μm). Asterisk marks imaged spine. (b_2_) Averaged baseline bAP-Ca^2+^ transients 0 to 5 min after bAP stimulation onset (grey) and averaged bAP-Ca^2+^ transients 15 to 20 min after bAP stimulation onset (blue) of a spine in the presence of 100 μM ryanodine. (b_3_) Time plot of normalized doublet evoked bAP-Ca^2+^ transient enhancement in spines with small baseline amplitudes comparing control (black) spines to spines in the presence of 100 μM ryanodine (blue). One doublet was applied every 60 s, the data is plotted in 3 min bins. (c_1_) Z-projection of the imaged spine segment (scale bar corresponds to 2 μm). Asterisk marks imaged spine. (c_2_) Averaged baseline bAP-Ca^2+^ transients 0 to 5 min after bAP stimulation onset (grey) and averaged bAP-Ca^2+^ transients 15 to 20 min after bAP stimulation onset (green) of a spine in the presence of 10 μM Xestospongin-C. (c_3_) Time plot of normalized doublet evoked bAP-Ca^2+^ transient enhancement in spines with small baseline amplitudes comparing control (black) spines to spines in the presence of 10 μM Xestospongin C (green). One doublet was applied every 60 s, the data is plotted in 3 min bins. (d_1_) Bar graph of normalized enhancement 15 to 20 min after bAP stimulation onset for spines with small baseline amplitudes in control spines (black, +33 ± 6%, *n* = 53/31 spines/cells), CPA (red, 0 ± 6%, *n* = 24/10 spines/cells), ryanodine (blue, -2 ± 7%, *n* = 27/16 spines/cells) and Xestospongin C (green, +14 ± 4%, *n* = 56/18 spines/cells). In comparison to the control group, CPA and ryanodine blocked the enhancement (*p* < 0.01 and *p* < 0.001, respectively), whereas enhancement was not significantly reduced by Xestospongin C (n.s., Kruskal-Wallis test with Dunn’s posthoc correction). (d_2_) Cumulative distribution plot of normalized bAP-Ca^2+^ transient enhancement. Dataset corresponds to d_1_. Data are expressed as mean SEM ** *p* < 0.01, *** *p* < 0.001.

Previous work demonstrated that VGCC mediated Ca^2+^ transients can directly activate the RyR intracellular Ca^2+^ release channel [24]. We hypothesized that the observed activity-dependent enhancement constitutes a new functional role for intracellular Ca^2+^ release from RyRs in spines. Depletion of intracellular Ca^2+^ stores by pre-incubation with 30 μM of CPA indeed blocked activity-dependent enhancement of bAP-Ca^2+^ transients (Figs 6A and 6D and S5A). To directly inhibit the RyR, we used 100 μM of ryanodine. At this concentration, ryanodine blocks the channel rather than locking it in a subconductance state [25,26]. Pre-incubation with ryanodine also inhibited the activity-dependent enhancement of bAP-Ca^2+^ transients (Figs 6B and 6D and S5B). We also investigated whether IP3Rs, the other family of intracellular Ca^2+^ release channels [27], are involved in enhancement. Using the IP3R antagonist Xestospongin C (10 μM) in the pipette, enhancement was not significantly affected when compared to controls (Figs 6C and 6D and S5C).

The full effect size of CPA on enhancement could be matched with the specific RyR antagonist ryanodine and the reduction of enhancement observed with Xestospongin C (which has been demonstrated to affect VGCCs in mammalian preparations [28]) did not reach statistical significance when compared to controls. Thus, our results demonstrate that bAP-Ca^2+^ transient enhancement depends on RyR mediated Ca^2+^ release from intracellular stores.

### Store Release Is Important for Induction but Not Expression of bAP-Ca^2+^ Transient Enhancement

**Fig 7:**
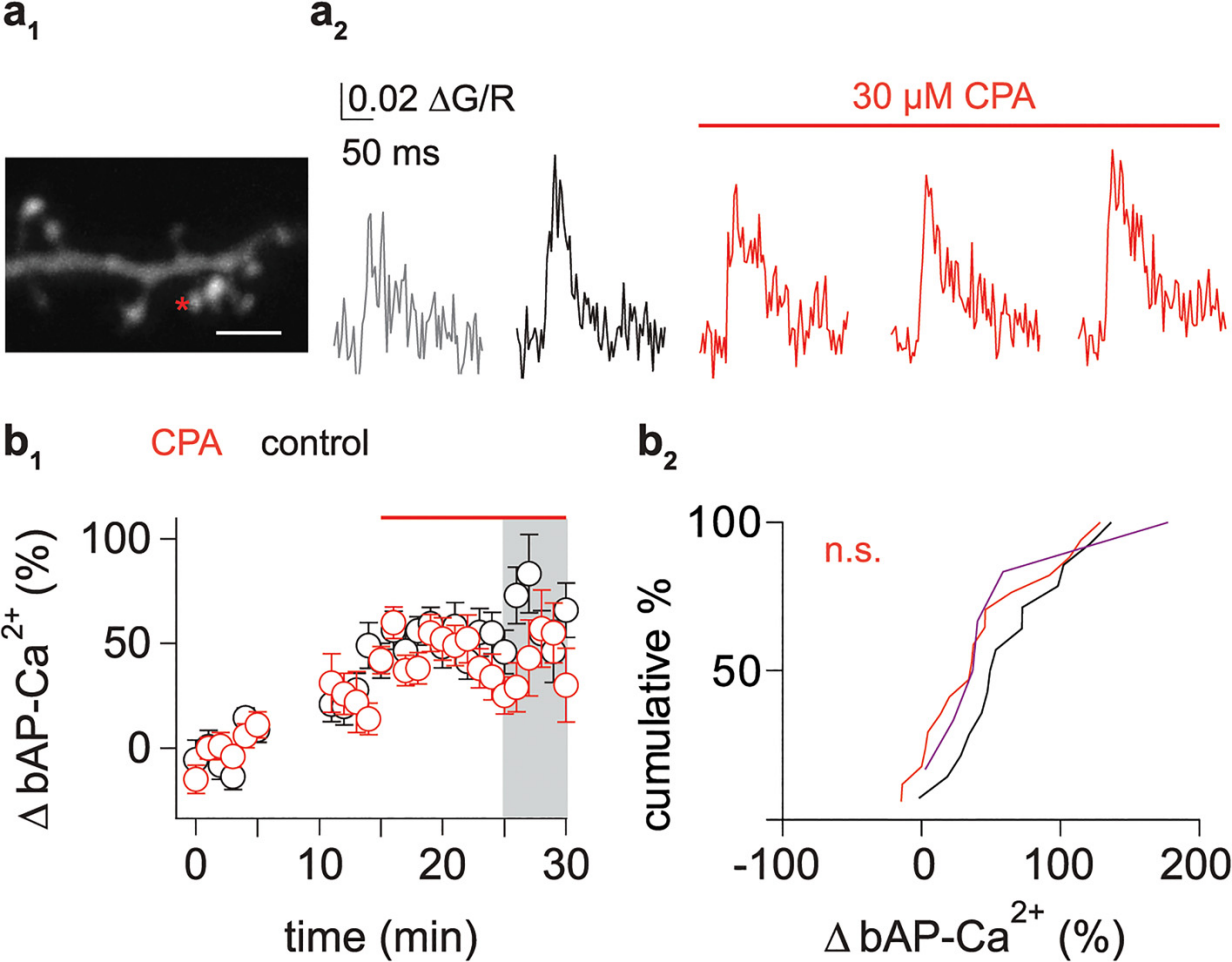
Stores play a role in induction but not in expression of bAP-Ca^2+^ transient enhancement. (a_1_) Z-projection of the imaged dendritic segment (scale bar corresponds to 2 μm). Asterisk marks imaged spine. (a_2_) Averaged traces of spine bAP-Ca^2+^ transients: baseline (grey, 0 to 5 min after onset of the experiment), pre-CPA wash-in (black, 10 to 15 min after onset), and post-CPA wash-in (red, 16 to 20, 21 to 25 and 26 to 30 min after the onset of the experiment). Red line indicates wash-in of 30 μM CPA. (b_1_) Time plot of preselected enhanced spines (enhancement >20% between 15 and 20 min after starting the experiment) comparing controls (black) and spines with CPA wash-in (red) 15 min after the onset of the experiment. Interval used for comparison between control spines and spines where CPA was washed in is shaded in grey. (b_2_) Cumulative distribution plot of normalized bAP-Ca^2+^ transient enhancement in time-matched controls and 10 min after CPA and combined CPA and ryanodine wash-in in spines (corresponds to grey area in b_1_). Control spine enhancement (black, +63 ± 11%, *n* = 14/10 spines/cells) is not significantly different from spines treated with 30 μM CPA alone (red, +42 ± 11%, *n* = 17/13 spines/cells, *p* = 0.07, one-tailed Mann Whitney U test). Combined treatment with CPA and ryanodine was tested in a small population of spines (+57 ± 23%, *n* = 6/3 spines/cells, magenta). Data are expressed as mean SEM.

We next asked whether the contribution of stores to bAP-Ca^2+^ transient enhancement results from store activation during induction or whether store activation underlies the expression of enhancement. To test the hypothesis that the expression of bAP-Ca^2+^ transient enhancement is mediated by recruitment of intracellular Ca^2+^ stores, 30 μM CPA was washed in after 15 min. This permitted to assess enhancement during our defined time interval for quantification of enhancement (Fig 3C) without an appreciable effect of CPA, as Fig 1C demonstrates that the first 5 min of CPA wash-in did not have an effect. We preselected for enhanced spines (enhancement >20% between 15 and 20 min after starting the experiment; as doublet application has been demonstrated to be sufficient in inducing enhancement, some enhancement can already be observed at the end of our 6 min baseline measurement when only the enhanced spines are plotted). In enhanced spines, 10 min of CPA application resulted in a small reduction of enhancement which was not significant when compared to enhanced control spines in the same time interval (Fig 7B). From Fig 1C, we can see that this 10 min wash-in of CPA already has a significant effect on the bAP-Ca^2+^ transient measured with 500 μM fluo-5F (CPA: -18 ± 3%, *n* = 17/3 spines/cells; control: +6 ± 5%, *n* = 13/3 spines/cells, *p* < 0.01, Mann Whitney U test). In a smaller subset of spines, we also washed in a combination of CPA and ryanodine aiming at a stronger inhibition of store release. This resulted in no appreciable reduction compared to controls (Fig 7B). The small trend towards reduction by CPA corresponds to the effect size observed in Fig 1, but cannot explain the expression level of enhancement reached in our experiments. We conclude that RyR mediated Ca^2+^ release from intracellular stores is a requirement during induction, but is not involved in the expression of enhancement.

### bAP-Ca^2+^ Transient Enhancement Is a Nanodomain Function of Spine RyRs

Spine RyRs contribute relatively little to the volume-averaged Ca^2+^ signal (Figs 1C and 7). This contribution could not be resolved by CPA wash-in in spines that undergo RyR-dependent enhancement using the low-affinity Ca^2+^ indicator fluo-4FF (Fig 7). At the same time, this small reduction of the volume-averaged signal results in block of bAP-Ca^2+^ transient enhancement (Fig 6). Ca^2+^ buffering by 500 μM fluo-5F still enables RyR activation (Fig 1), but reduces enhancement significantly (Fig 5D). Taken together, these findings suggest that RyRs are coupled to a Ca^2+^ activated effector in a specific intraspine Ca^2+^ signalling domain. Effector activation in this domain would mediate bAP-Ca^2+^ transient enhancement spatially and functionally uncoupled from Ca^2+^ influx at the plasma membrane.

**Fig 8:**
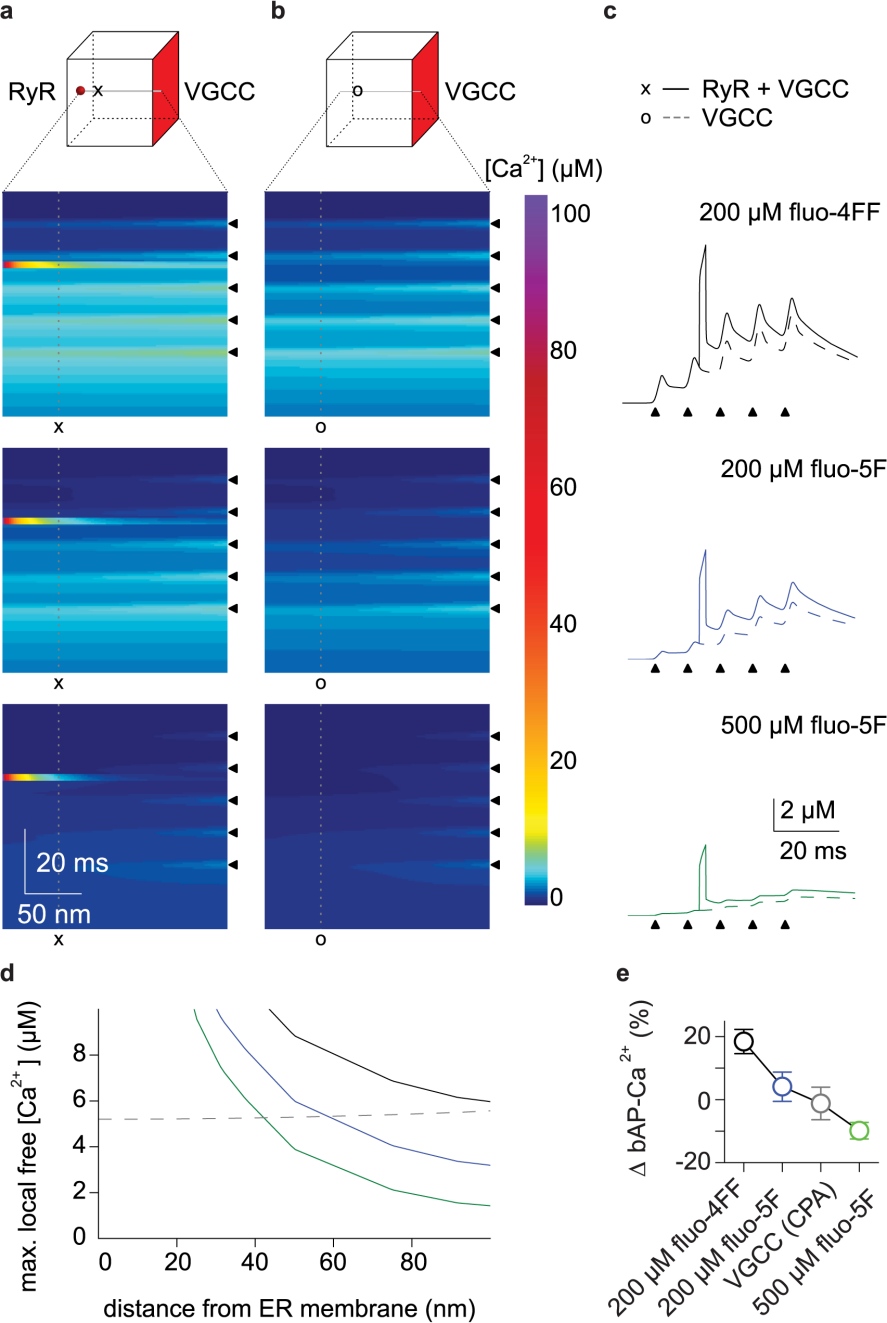
Modelling of RyR mediated intraspine Ca^2+^ nanodomains. (a) Simulated linescan of the time course of [Ca^2+^] along a 200 nm line between the RyR located within the spine and VGCCs distributed across the opposite membrane surface. Black triangles correspond to bAPs in a 5 AP burst. Simulations were run with 200 μM fluo-4FF (top), 200 μM fluo-5F (middle) and 500 μM fluo-5F (bottom) as buffers. (b) Same as in (a) with RyRs omitted (VGCCs only). (c) [Ca^2+^] over time at a distance of 50 nm from the RyR channel pore (lines marked with x and o in a and b). Straight lines correspond to a scenario with RyRs and VGCCs, dotted lines to a scenario with VGCCs only. Different colours correspond to 200 μM fluo-4FF (black, top), 200 μM fluo-5F (blue, middle), and 500 μM fluo-5F (green, bottom) as buffers. (d) Spatial distribution of [Ca^2+^] maxima after 5th bAP with VGCC activation only (dotted line) or after RyR opening following 2nd bAP (solid lines) under different buffering conditions: 200 μM fluo-4FF (black), 200 μM fluo-5F (blue) and 500 μM fluo-5F (green). (e) Effect sizes. Plot of normalized bAP-Ca^2+^ transient enhancement 15 to 20 min after stimulation onset in all spines measured under different conditions. Black: 200 μM fluo-4FF, +19 ± 4%, *n* = 92/43 spines/cells; blue: 200 μM fluo-5F, +4 ± 5%, *n* = 31/7 spines/cells; grey: CPA (VGCCs only), -1 ± 5%, *n* = 31/11 spines/cells; green: 500 μM fluo-5F, -10 ± 3%, *n* = 68/24 spines/cells.

Ca^2+^ signalling domains within a spine cannot be temporally and spatially resolved with two-photon or confocal Ca^2+^ imaging. We therefore modelled spine Ca^2+^ dynamics. Our results were consistent with the idea that RyR Ca^2+^ domains trigger the activation of downstream effectors. We assumed a three-dimensional domain with membrane channels on one side and a single RyR on the opposite side (200 nm distance, Fig 8A and 8B). Reaction-diffusion equations for release and buffered diffusion of Ca^2+^ in the spine were numerically solved for given sequences of Ca^2+^ influx. APs led to opening of evenly distributed voltage-gated channels in the membrane, which in turn raised intracellular Ca^2+^ concentrations to about 1 μM. Although existing kinetic models of the RyR guarantee the dynamic opening of the receptor channel by Ca^2+^ concentrations in this range, we here implemented opening of this single RyR after doublet firing by default.

We then compared the spatiotemporal profile of free intraspine [Ca^2+^] in the presence of 200 μM fluo-4FF, 500 μM fluo-5F, and 200 μM fluo-5F (Fig 8A, 8C and 8D). In addition, we compared the spatiotemporal distribution of intraspine ∆[Ca^2+^] in spines with a homogenous distribution of VGCCs on the plasma membrane (Fig 8B, 8C and 8D) to that in spines where we added a single RyR 200 nm away from the plasma membrane. In our model, release through the RyR established a nanodomain around the channel. The spatial profiles of this nanodomain are plotted in Fig 8D for the different buffering conditions added by the Ca^2+^ indicators. Induction of enhancement is blocked in CPA and ryanodine (Figs 6, 7 and 8E). Pharmacological block of RyRs results in Ca^2+^ transients exclusively mediated by VGCCs. Enhancement block in CPA and ryanodine therefore demonstrates that RyR mediated increases in ∆[Ca^2+^] cannot be compensated by VGCC activation. This demonstrates specificity of Ca^2+^ signalling within the RyR nanodomain. The spatial profile of [Ca^2+^] mediated by exclusive VGCC activation is represented by the dotted line in Fig 8D. This enabled us to identify a spatial window around the RyR pore where the RyR mediated ∆[Ca^2+^] was larger than the maximal intraspine ∆[Ca^2+^] mediated by VGCC influx alone (intersection of the RyR nanodomain's spatial profile with the dotted line in Fig 8D). In addition, excess buffering mediated by 500 μM fluo-5F prevented effector activation to a larger degree than store block, whereas partial activation was achieved at 200 μM fluo-5F (Fig 8E). This further constrained the coupling distance between the RyR and the Ca^2+^ activated effector. Our experimental and modelling results suggest that the potentiating effector is located within approximately 90 nm from the RyR and should have a K_d_ larger than 5 μM (Fig 8D).

## Discussion

We demonstrate electrochemical coupling between bAPs and intracellular Ca^2+^ release from stores in dendritic spines. On a functional level, bAP mediated activation of spine RyRs results in an activity-dependent enhancement of the bAP-Ca^2+^ transient, namely a compartmentalized plastic change of a spine's functional state. Enhancement is independent of plasticity dynamics in adjacent spines and dendritic segments. It also scales with neuronal suprathreshold activity levels. In spite of a relatively small contribution of RyRs to the volume-averaged spine Ca^2+^ transient, RyRs form a functional Ca^2+^ nanodomain inside the spine that specifically induces bAP-Ca^2+^ transient enhancement without contributing to the expression of the enhanced signal.

Previous work on plasticity of bAP-Ca^2+^ transients in dendritic spines of CA1 pyramidal neurons demonstrated activity-dependent depression. The authors did not detect changes in dendritic bAP-Ca^2+^ transients and described a reduction in spine R-Type Ca^2+^ channels [29]. Under different conditions (lower Ca^2+^-indicator induced buffer capacity, milder stimulus protocol), we observed activity-dependent enhancement of bAP-Ca^2+^ transients, suggesting bidirectional plasticity. Neuromodulatory pathways for the induction of plastic bAP-Ca^2+^ transient changes have already been described. Pharmacological activation of ß_2_ adrenergic receptors has been shown to specifically enhance L-type Ca^2+^ channels in dendritic spines [30]. Selective reduction of spine bAP-Ca^2+^ transients upon pharmacological GABA_B_ receptor activation was demonstrated in CA1 pyramidal cell basal dendrites [31], most likely by direct inhibition of spine VGCCs [32]. Together, these results suggest a compartment specific state-dependent bidirectionality of activity-dependent changes in bAP-Ca^2+^ transients. These spine specific changes in bAP-Ca^2+^ transient size reported so far have been expressed by direct modulation of the number and/or permeability of VGCCs on the plasma membrane.

Alternatively, spine-specific changes in electrical properties mediated by specific expression patterns of K^+^ channels [33,34] or the spine neck resistance [35,36] could mediate the observed effect. Are electrical factors likely to contribute to spine-specific enhancement of bAP-Ca^2+^ transients? Synaptic activation occurs in the spine head and travels into the dendrite. High input impedance of the serially arranged spine head and neck results in clear compartmentalization of these synaptically evoked electrical signals arising in the spine head [37–41]. However, bAPs travel in the other direction, from the dendrite to the spine. Both bAPs as well as slow dendritic voltage changes have been predicted and experimentally shown to be virtually unchanged by the electrical properties of the spine [42–44], but see [45]. Changes in a spine’s electrical properties are, therefore, unlikely candidates to mediate compartmentalized bAP induced Ca^2+^ signal plasticity; based on the impedance mismatch between spines and dendrites, they are likely to be overridden by the intrinsic properties of the adjacent dendritic segment.

Our post-induction wash-in experiments with CPA and ryanodine rule out a significant contribution of intracellular stores to the expression of bAP-Ca^2+^ transient enhancement. We conclude that, in line with previous studies, the expression of enhancement described here is based on modulation of VGCCs. Future work will have to analyse how VGCCs are amplified during enhancement.

Functional consequences of enhancement are likely linked to functions of bAP-Ca^2+^ transients described so far. bAPs transmit information about neuronal output into the dendritic tree and the adjacent spines. By interacting with active conductances as well as AMPA and NMDARs, this additional depolarization can have nonlinear effects resulting in changes in both short- and long-term synaptic plasticity [46]. The bAP-mediated depolarization does not only affect other active conductances but is accompanied by spine and dendritic Ca^2+^ influx via VGCCs. This Ca^2+^ influx may contribute to the induction of synaptic plasticity [29]. Less is known about the functional role of bAP-Ca^2+^ transients in spines and dendritic segments when there is no interaction with coincident synaptic inputs. There is evidence for Ca^2+^ induced, non-hebbian synaptic plasticity induced by postsynaptic AP firing only [47,48]. In addition, AP firing preceding the induction of long term potentiation (LTP) has been demonstrated to enhance LTP, linking these signals to metaplasticity [49]. A recent study in juvenile rats reports dependence of spine shrinkage and long term depression (LTD) on the bAP-Ca^2+^ transient amplitude: Above a certain bAP mediated spine [Ca^2+^], spines are resistant to shrinkage and LTD [50].

We demonstrate a direct contribution of RyR mediated Ca^2+^ release to the bAP-Ca^2+^ transient, but this effect is small and does not underlie the expression of enhancement. We explore how this relatively small contribution to the bulk Ca^2+^ signal can be functionally relevant. Our data supports an important functional contribution of RyR mediated store release to the dynamics of bAP-Ca^2+^ transients: The induction block of bAP-Ca^2+^ transient enhancement by store depletion and RyR block, the small but significant contribution of store release to bAP-Ca^2+^ transients and our modelling data suggest that RyRs can form specific functional nanodomains in the spine's cytosol. In line with this, the large cytoplasmic portion of RyRs serves as a scaffold forming a macromolecular signalling complex with a plethora of different associated proteins [51]. Modelling permits us to make assumptions about the downstream effector mediating enhancement, which is tightly coupled to the RyR in a range of up to 90 nm and has a K_d_ for Ca^2+^ in the range of 5 μM or more. Considering the narrow window between 500 μM fluo-5F and 200 μM fluo-4FF with respect to the free nanodomain [Ca^2+^], Ca^2+^ binding to the effector is most likely cooperative. We observe enhancement of the bAP-Ca^2+^ transient predominantly in spines with small baseline amplitudes. This suggests that there is a fixed dynamic range for bAP mediated spine [Ca^2+^], which limits further enhancement in spines where the downstream effector has already been activated. In this context, it is an interesting observation that we can induce enhancement in the small baseline amplitude spines over a wide range of small amplitude doublets, but fail to do so with single bAPs. One explanation is that doublets will prolong the time window of RyR activation (S1 Fig). The necessity for doublet firing therefore suggests that the lifetime of the RyR Ca^2+^ nanodomain critically determines downstream effector activation.

Functionally, a specific RyR gated Ca^2+^ signalling nanodomain can be regulated independent fromVGCCs at the plasma membrane. In contrast to VGCC activation by bAPs, the recruitment of intracellular stores is a biochemical property independent of the electrical properties of the adjacent dendritic segment. Ca^2+^ nanodomains tightly coupled to RyRs are therefore ideally suited to implement a compartmentalized spine-specific Ca^2+^ code. The RyR and its associated proteins have an abundance of potential interaction sites with other signal transduction pathways [51]. This way, RyR mediated nanodomain signalling in spines contributes to the versatility of neuronal Ca^2+^ signalling, further empowering a single second messenger molecule to encode many different cellular responses. Nanodomain coupling without a small contribution to the bulk Ca^2+^ signal likely resulted in underestimation of the significance of Ca^2+^ induced Ca^2+^ release via intracellular stores in spine Ca^2+^ signalling in previous studies (for review, see [14,27]).

A contribution of nanodomain signalling of RyRs has to be considered for a number of physiological and pathophysiological processes during synaptic plasticity and memory: RyRs are up-regulated during intense training tasks [52] and mediate Brain Derived Neurotrophic Factor (BDNF) related effects on plasticity and memory [53]. They are involved in metaplastic priming of synapses for long term potentiation [54]. On a pathophysiological level, RyRs contribute to stress-induced hippocampal cognitive dysfunction [55]. Hyper-reactivity of spine RyRs has been demonstrated to precede the onset of symptoms in Alzheimer disease [56]. It will be interesting to find out if the new functional role of RyRs we discovered in layer 2 of the MEC is linked to the early onset of Alzheimer-disease–related dysfunction in this brain region [57].

In addition to demonstrating that RyRs can form specific intraspine signalling nanodomains, we also propose a new function for intracellular Ca^2+^ release in neurons: RyRs can induce a plastic functional state change in spines that scales with neuronal output levels. This compartmentalized storage of global activity levels provides a new mechanism of how endoplasmic reticulum mediated Ca^2+^ release contributes to neuronal information processing.

## Materials and Methods

### Slice Preparation

Animal experiments were performed in accordance with institutional and national guidelines. Acute cortical slices were prepared from Wistar rats (age: postnatal day 16–25). Animals were anesthetized with isoflurane and decapitated. See [58] for details.

For pre-incubation experiments, 30 μM CPA or 100 μM ryanodine or a synaptic receptor inhibitor cocktail consisting of the NMDAR antagonist 2R-amino-5-phosphonovaleric acid (APV, 100 μM) the AMPA and Kainate receptor antagonist 2,3-dihydroxy-6-nitro-7-sulfamoyl-benzo[f]quinoxaline-2,3-dione (NBQX, 20 μM) and the group I metabotropic glutamate receptor (mGluR) antagonist (S)-α-Methyl-4-carboxyphenylglycine ([S]-MCPG), 500 μM—substituted by 2-Methyl-6-(phenylethynyl)pyridine, (MPEP), 25μM and (S)-(+)-α-Amino-4-carboxy-2-methylbe­nzeneacetic acid (LY 367385), 100 μM, in some experiments—as well as the GABA_A_R antagonist Gabazine (1 μM) was added to the ACSF. Slices were preincubated in the respective drug solution for at least 30 min before starting the experiment. Control experiments were interleaved with experiments where drugs were applied. Wash-in experiments were performed with 30 μM CPA or a mixture of 30 μM CPA and 10 μM ryanodine to facilitate store depletion.

### Electrophysiology

Whole-cell current clamp experiments were performed at near physiological temperature (32–34°C) using an Axon Multiclamp 700B amplifier (Molecular Devices, Sunnydale, CA, US). Signals were low pass filtered at 2 kHz and digitized at a sampling rate of 5 kHz (BNC-2090, National Instruments Corporation, Austin, Tx, US). Pipettes (3–6 MΩ) were filled with an intracellular solution containing 130 K-gluconate, 20 KCl, 10 HEPES, 4 MgATP, 0.3 NaGTP and 10 phosphocreatine (in mM; pH: 7.3) and 30 μM Alexa 594 and 200 μM fluo-4FF or 200 μM fluo-5F or 500 μM fluo-5F. Initial series resistances were between 6 and 20 MΩ. Action potentials were induced with 2 ms square current pulses ranging from 1 to 3 nA. Doublets and quintuplets were delivered at 100 Hz. Experiments were aborted if the holding current exceeded -200 pA at -60 mV.

### Two-Photon Calcium Imaging

A Femto2D two-photon laser scanning system (Femtonics Ltd., Budapest, Hungary) equipped with a femtosecond pulsed Ti:Sapphire laser tuned to λ = 805 nm (Cameleon, Coherent, Santa Clara, CA, United States) controlled by the Matlab-based MES software package (Femtonics Ltd., Budapest, Hungary) was used. Fluorescence was detected in epifluorescence mode with a water immersion objective (LUMPLFL 60x/1.0 NA, Olympus, Hamburg, Germany). Transfluorescence and transmitted infra-red were detected using an oil immersion condenser (Olympus).

We filled the cells for at least 25 min with dye before multiple line-scans of dendritic spines and the adjacent dendritic segment were taken [22]. The average scanning speed was 300 Hz and the intermediate sections were jumped over within 60 μs using a spline interpolated path. bAP-Ca^2+^ transients of the doublet test stimulus were measured every 60 s. After six baseline sweeps, we delivered bursts of 5 APs at 100 Hz every 30 s for 5 min (see Fig 2A). We did not perform imaging experiments during burst delivery. After ten 5 AP bursts, the test stimulus was again delivered every 60 s starting 1 min after the last quintuplet for 15 to 20 min. Imaging of the test stimulus was taken up again 1 or 5 min after induction.

Calibration of Gmax/R values was performed at the tip of a sealed pipette in the imaging plane of the slice using a calibration solution consisting of 50 μl recording solution and 50 μl 1M Ca^2+^Cl^-^. Several batches of recording solution were measured at different time points (*n* = 30 for fluo-4FF, *n* = 9 for fluo-5F). For morphological reconstructions, we performed post-hoc high-resolution z-stacks of the recorded spines with a ∆z of 0.2 μm.

### Data Analysis and Statistics

Changes in [Ca^2+^] are reported as the ratio of green fluorescence change over red (∆G/R) as described by Yasuda et al. [23]. For quantification of bAP-Ca^2+^ amplitudes, ∆G/R was averaged over a 70 ms time interval starting with the first AP. The analysis interval was chosen because we often observed bAP-Ca^2+^ transients that exhibited a second, slow component after the bAP-related peaks (see for example Fig 2B_2_, 16 to 20 min). The 70 ms interval was defined based on visual inspection of the data for maximising effect size of enhancement and the signal-to-noise ratio. As a control, we also performed our analysis on a shorter 20 ms time interval starting after the second AP to focus on the peak of the Ca^2+^ signal. Levels of enhancement in both time windows were strongly correlated (r = 0.85, *p* < 0.0001, S6 Fig) and not significantly different from each other (Mann Whitney U test).

To exclude line scan measurements with spines out of focus, measurements were excluded when the Alexa 594 intensity was below 80% of the average baseline intensity. A further exclusion criterion was the rise in the background corrected baseline green over red (G_0b_R) above 20% of the average baseline value in three consecutive sweeps. Increases in G_0_R indicate a rise in baseline Ca^2+^ suggesting a deterioration in cell health [23]. Pre-induction ∆G/R amplitudes are averages of three to six sweeps.

The 15 min stimulation period prior to our time interval for quantification of enhancement was structured as follows: A 6 min baseline of six doublets (2 APs at 100 Hz, interdoublet interval 60 s) was followed by 10 bursts of 5 APs (100 Hz, 30 s interburst interval). After this 5 min bursting period, we again switched to doublets. The 15 min stimulation period was completed by a 4 min application of four doublets post-burst. After this 15 min stimulation period, we quantified the spine-specific enhancement as a post/pre percentage change of the doublet bAP-Ca^2+^ transient for each individual spine. In detail, we divided the averaged doublet amplitude, taken 15 to 20 min after the onset of bAP stimulation, by the averaged amplitude of the baseline doublets recorded in the first 6 min after the onset of stimulation. Changes were then displayed as ∆%.

Spines were only included if at least three out of six sweeps in the pre- and post- time interval fulfilled the above-mentioned criteria for focus and cell health. In addition, in order to form a meaningful post-/pre- percentage ratio as well as to determine a time constant τ we defined an inclusion criterion for the signal-to-noise ratio (S/N) of the pre-induction signal. In averaged traces of the pre-induction sweeps, the amplitude averaged over 20 ms after the second AP in a doublet had to be 2.5 times larger than the standard deviation of a 40 ms pre-AP baseline stretch.

Unless otherwise noted, statistical comparisons of enhancement were performed between grouped spine populations with small baseline doublet bAP-Ca^2+^ transient amplitudes. Baseline doublet application already has an effect on enhancement measured at a later time point (Fig 4D). For grouping of single AP data, we, therefore, applied test doublets after the last single AP. These test doublets permitted us to group spines that had previously only been stimulated with single APs into spines which showed a small or a large bAP-Ca^2+^ transient in response to doublets. This grouping enabled us to statistically compare single bAP-Ca^2+^ transient enhancement with the doublet-based control group of spines with small baseline bAP-Ca^2+^ transients.

Head size and spine length were estimated from maximum intensity projections of z-stacks of the spines and the adjacent dendritic segment. The apparent spine size was approximated by measuring the FWHM of the maximal spine diameter x. The diameter of spines is below the resolution limit of a 2-photon microscope. We, therefore, implemented a correction factor k by dividing the maximal brightness of a spine by the maximal brightness of the adjacent dendritic segment. This correction is based on the assumption that the dendritic segment is larger than the resolution limit [59]. Spine length was determined from the origin of the spine at the dendrite to the middle of the spine head.

For statistical comparison, student's *t* test or ANOVA with Bonferroni correction were used for normally distributed data. Normality was tested using the Shapiro Wilk normality test. If there was no normal distribution, Mann Whitney U test or the Kruskal Wallis test with Dunn's posthoc correction were used as indicated. When testing against a theoretical change of 0%, one sample *t* tests were used for normally distributed data and Wilcoxon signed rank tests were used for non-normally distributed data. Correlations were tested using Spearman's rank order test. Significance level for all statistical tests was at *p* < 0.05. All averages are reported as mean ± SEM.

### Modelling

#### Deterministic reaction-diffusion system

Three-dimensional reaction-diffusion equations are used to describe the concentration fields of Ca^2+^, Ca^2+^-indicator, and endogenous buffers. [Ca^2+^] sources and sinks, such as VGCC, RyR, SERCA, PMCA, and outflow of [Ca^2+^] through the spine neck are taken into account by specific boundary conditions and additional source terms in the equations. In the following, the free cytosolic [Ca^2+^], bound endogenous buffer and bound dye buffer are denoted as *c*, *b*_*e*_, and *b*_*d*_, respectively. The set of reaction-diffusion equations reads:

$$\begin{array}{l} \;\frac{\partial c}{\partial t}=D_{c}\nabla^{2}c+b_{e}k_{e}^{-}-c\;k_{e}^{+}\left(B_{e}-b_{e}\right)+b_{d}k_{d}^{-}-c\;k_{d}^{+}\left(B_{d}-b_{d}\right)-\lambda \left(c-c_{0}\right),\\ \frac{\partial b_{e}}{\partial t}=D_{e}\nabla^{2}b_{e}-b_{e}k_{e}^{-}+c\;k_{e}^{+}\left(B_{e}-b_{e}\right),\\ \frac{\partial b_{d}}{\partial t}=D_{d}\nabla^{2}b_{d}-b_{d}k_{d}^{-}+c\;k_{d}^{+}\left(B_{d}-b_{d}\right). \end{array}$$

*B*_*e*,*d*_ denote the total buffer concentrations. The on- and off-rates of buffers and their diffusion coefficients are denoted as $k_{e,d}^{+}$, $k_{e,d}^{-}$, and *D*_*e*,*d*_, respectively. The resting [Ca^2+^] and the free calcium diffusion coefficient are denoted as *c*_0_ and *D*_*c*_, respectively. Ca^2+^ extrusion caused by efflux through the spine neck and PMCA is modelled by the term −*λ*(*c*−*c*_0_), a homogeneous [Ca^2+^] sink where *λ* determines the time scale of the extrusion. The above equations for free [Ca^2+^], dye and endogenous buffers are solved in a rectangular box representing the spine head. Calcium transport through the ER membrane ∂Ω_ER_ (left side in Fig 8A and 8B) and the plasma membrane ∂Ω_PM_ (right side) is modelled by inhomogeneous Neumann boundary conditions:

$$\begin{array}{l} D_{c}\;\vec{n}\vec{\nabla }c=P_{RyR}S\left(\vec{r},t\right)\left(E_{0}-c\right)-P_{SERCA}\frac{c^{2}}{K_{SERCA}^{2}+c^{2}}+P_{leak}\left(E_{0}-c\right)\;\text{at}\;\partial \Omega_{\text{ER}},\\ D_{c}\;\vec{n}\vec{\nabla }c=A_{0}\frac{1}{\sigma \sqrt{2\pi }}\sum_{i=0}^{N}\exp\left[-\frac{{\left(t-i\;\tau \right)}^{2}}{2\sigma^{2}}\right]\;\text{at}\;\partial \Omega_{\text{PM}}. \end{array}$$

Here, $\vec{n}$ denotes the unit outer normal on the boundary. *P*_*RyR*_, *P*_*SERCA*_, and *P*_*leak*_ control the maximum effect size of those three calcium fluxes. $S\left(\vec{r},t\right)$ evaluates to either 0 or 1, depending on whether the RyR is open at time *t* and whether $\vec{r}(t)$ is within the pore disc of radius *R*_*c*_ around the channel location. *K*_*SERCA*_ denotes the dissociation coefficient of SERCA pumps. To obtain a resting state with given free Ca^2+^ concentrations *c*_0_ (cytosol) and *E*_0_ (endoplasmic reticulum), *P*_*leak*_ has to obey:

$$P_{leak}=\frac{P_{SERCA}\;c_{0}^{2}}{\left(E_{0}-c_{0}\right)\left(K_{SERCA}^{2}+c_{0}^{2}\right)}.$$

The openings of VGCCs triggered by a given sequence of bAPs are represented as train of *N* identical Gaussian pulses (width *σ*) separated by a delay *τ*. *A*_0_ controls the number of Ca^2+^ ions that enter per bAP. In simulations that include RyR efflux, we assumed that a single RyR will open for 2 ms after the second bAP, i.e., $S\left(\vec{r},t\right)=1$ for *t* ∈ [0.112*s*,0.114*s*]. For the deduction of the parameters introduced above, please see S1 Table.

The set of reaction-diffusion equations was numerically solved using the DUNE open source software library for finite element calculations [60–62]. The spatial domain was discretised by a locally refined conforming grid where the grid resolution gradually increased from 3 nm at the channel pore to 50 nm at the outer spine membrane. Time integration was performed with a three stage linear implicit Runge-Kutta algorithm with embedded error estimator which was used to deduce adaptive time steps (ranging from 10 ns during VGCC influx and RyR channel openings to 100 ms for the following calcium concentration decay).

#### Ryanodine receptor model

The RyR gating model is based on the model from Sobie et al. [63,64]. Here, a single channel is subjected to stochastic transitions between its open and closed state. While the close rate is constant, the open rate depends on both cytosolic and luminal [Ca^2+^]. The open and close rates *k*_*o*_ and *k*_*c*_ of a channel are given in the following way:

$$\begin{array}{l} k_{o}=\rho_{+}\frac{c^{4}}{c^{4}+{\left[\alpha \left(K_{m}-E\right)\right]}^{4}},\\ k_{c}=\rho_{-}, \end{array}$$

where *c* and *E* denote cytosolic and luminal free [Ca^2+^], respectively. The stationary open probability of a single RyR channel is given by

$$P_{o}=\frac{1/k_{c}}{1/k_{c}+1/k_{o}}=\frac{\rho_{+}}{\rho_{+}+\rho_{-}}\;\frac{c^{4}}{c^{4}+K_{d}^{4}}$$

with

$$K_{d}=\left[\alpha \left(K_{m}-E\right)\right]{\left(\frac{\rho_{-}}{\rho_{+}+\rho_{-}}\right)}^{1/4}.$$

The parameter values that we use can be found in S1 Table. The *α* and *K*_*m*_ parameters have been changed from the values provided in [64] to obtain a binding constant of *K*_*d*_ ≈ 1 μM (for a full ER with *E*_0_ = 700 μM), which is in agreement with single channel measurements [65]. Given *N* closed channels at *t* = 0, the probability density *p* and the cumulative density function *F* of *p* for the opening of any of the *N* channels at time *t* are:

$$\begin{array}{l} p\left(t\right)=N\;k_{o}\left(t\right)\exp\left[-\overset{t}{\underset{0}{\int }}ds\;N\;k_{o}\left(s\right)\right],\\ F\left(t\right)=1-\exp\left[-\overset{t}{\underset{0}{\int }}ds\;N\;k_{o}\left(s\right)\right]. \end{array}$$

## Supporting Information

S1 Data Numerical data underlying panels in the figures corresponding to the datasheets as indicated on the tabs of the excel sheet are provided. Please see the Methods section for how the data was analysed to generate these numerical values and where we define the quality criteria data points need to fulfil to be included in the analysis.
(XLSX)

S1 Fig Store activation by bAPs. (a_1_) Time plot of normalized changes in the time constant τ of doublet evoked bAP-Ca^2+^ amplitudes comparing control (black) and CPA wash-in (red) after 5 min of baseline. One doublet was applied every 60 s, the data is plotted in 3 min bins. Interval used for post-/pre- comparisons of changes in the time constant is shaded in grey. CPA wash-in resulted in a significant prolongation of τ by 22 ± 12% (*n* = 17/3 spines/cells) compared to control conditions (-8 ± 6%; *n* = 12/3 spines cells, *p* < 0.05, one-tailed Mann Whitney U test). (a_2_) Pairwise comparison of baseline decay time constants with time constants 15 to 20 min after CPA wash-in (red, *n* = 17/3 spines/cells) and time-matched control values (black, *n* = 12/3 spines/cells; *p* < 0.05 for CPA, *p* = 0.15 for controls, Wilcoxon signed rank test). (b) Ryanodine and CPA mediated reduction of bAP-Ca^2+^ transients are both a consequence of inhibited intracellular Ca^2+^ release. We, therefore, pooled the CPA and ryanodine data to further characterize the spines in which intracellular Ca^2+^ release was inhibited. Pooled CPA and ryanodine effect expressed as a normalized ratio 20 to 25 min after the onset of stimulation versus baseline plotted against apparent spine size, no significant correlation (r = 0.12, *n* = 36/8 spines/cells, *p* = 0.7, Spearman’s rank order test). (c) Pooled CPA and ryanodine effect expressed as a normalized ratio 20 to 25 min after the onset of stimulation plotted against baseline amplitude, significant negative correlation (r = -0.49, *n* = 37/8 spines/cells, *p* < 0.01, Spearman’s rank order test). (d_1_) Modelled time course of the VGCC mediated bAP-[Ca^2+^] for one AP (grey) and a doublet (black) at the Ca^2+^ activation site of the RyR pore in the presence of 500 μM fluo-5F. (d_2_) Time course of the cumulative open probability of a single RyR tetrameric channel activated by the VGCC mediated bAP-[Ca^2+^] changes plotted in d_1_. (e) Time course of the steady state open probability of a single RyR tetrameric channel activated by VGCC mediated bAP-[Ca^2+^] changes under conditions of reduced buffering in the presence of 200 μM fluo-4FF.
(EPS)

S2 Fig Basic properties of spines in CA1 and MEC. (a) Distribution histogram of baseline amplitudes for CA1 pyramidal cells (green) and layer 2 cells in the MEC (black). All baseline measurements in drug-free control conditions were included (MEC: *n* = 650/160 spines/cells; CA1: *n* = 189/42 spines/cells). Amplitudes in MEC were significantly larger than in CA1 (0.043 ± 0.001 ∆G/R versus 0.028 ± 0.001 ∆G/R, *p* < 0.0001, Mann Whitney U test). Using a Shapiro Wilk normality test, both datasets differed significantly from a normal distribution (MEC: *p* < 0.0001; CA1: *p* < 0.05). (b) Baseline amplitudes of all spine measurements in drug-free control conditions with recovered spine morphology for MEC (black, *n* = 582/155 spines/cells) and CA1 (green, *n* = 181/42 spines/cells). Amplitudes are plotted against the apparent size of the respective spine (MEC: r = -0.02, *p* = 0.69; CA1: r = -0.1, *p* = 0.2; Spearman’s rank order test). (c) Baseline amplitudes of all spine measurements in drug-free control conditions with recovered spine length for MEC (black, *n* = 622/157 spines/cells) and CA1 (green, *n* = 183/42 spines/cells). Amplitudes are plotted against the length of the respective spine (MEC: r = -0.06, *p* = 0.13; CA1: r = 0.04, *p* = 0.56; Spearman’s rank order test).
(EPS)

S3 Fig Spines predisposed for enhancement. (a) Spine distance from soma (μm) is plotted against normalized bAP-Ca^2+^ transient enhancement (r = 0.013, *n* = 79/37 spines/cells, *p* = 0.91, Spearman’s rank order test). (b) Apparent spine size is plotted against normalized bAP-Ca^2+^ transient enhancement (r = -0.013, *n* = 89/43 spines/cells, *p* = 0.9, Spearman’s rank order test). (c) Spine length is plotted against normalized bAP-Ca^2+^ transient enhancement (r = -0.04, *n* = 92/43 spines/cells, *p* = 0.72, Spearman’s rank order test). (d_1_) Averaged baseline bAP-Ca^2+^ transients 0 to 5 min after bAP stimulation onset (grey traces) and averaged bAP-Ca^2+^ transients 15 to 20 minutes after bAP stimulation onset of a spine (black) and the adjacent dendritic microdomain (red).(d_2_) Z-projection of the imaged dendritic segment (scale bar corresponds to 2 μm). Asterisks mark imaged spine and dendritic segment. (e) Normalized bAP-Ca^2+^ transient in spines 15 to 20 min after bAP stimulation onset is plotted against the corresponding dendritic microdomain (±1 μm). Values are not significantly correlated (*n* = 21/17 spines/cells, r = 0.35, *p* = 0.12, Spearman’s rank order test). Dashed lines correspond to enhancement >20%. Averaged values in spines are significantly larger than in dendrites (spines: 26 ± 8%, *n* = 21/17 spines/cells; dendrites: -10 ± 5%, *n* = 18/17 dendrites/cells, *p* < 0.001, paired *t* test). (f_1_) For CA1 pyramidal cells in the hippocampus, the averaged baseline amplitude is plotted against bAP-Ca^2+^ transient enhancement 15 to 20 min after bAP stimulation onset (r = -0.51, *n* = 59/22 spines/cells, *p* < 0.0001, Spearman’s rank order test). Spines with baseline amplitudes <0.026 ∆G/R are depicted in green (*n* = 17/8 spines/cells), spines with baseline amplitudes >0.026 are depicted in orange (*n* = 42/16 spines/cells). (f_2_) Time plot of normalized bAP-Ca^2+^ transients demonstrates selective enhancement of spines with baseline amplitudes <0.026 ∆G/R (green) when compared to spines with baseline amplitudes >0.026 ∆G/R (orange). Dendrites are plotted in red. One doublet was applied every 60 s, the data is plotted in 3 min bins. The interval 15 to 20 min after bAP stimulation onset used for quantification of normalized enhancement is shaded in grey. (f_3_) Cumulative distribution plot of normalized enhancement 15 to 20 min after bAP stimulation onset. Enhancement for spines with baseline ∆G/R < 0.026 (green, +24 ± 9%, *n* = 17/8 spines/cells) is significantly larger than in spines with baseline ∆G/R > 0.026 (orange, +1 ± 4%, *n* = 42/16 spines/cells, *p* < 0.05). The trend towards smaller enhancement of adjacent dendrites was statistically not significant. (red, +4 ± 6%, *n* = 19/19, dendrites/cells, n.s., ANOVA with Bonferroni's Multiple Comparison Test). Data are expressed as mean ± SEM, * *p* < 0.05
(EPS)

S4 Fig Scatter plots of enhancement using synaptic blockers and fluo-5F. (a) The averaged baseline amplitude is plotted against bAP-Ca^2+^ transient enhancement 15 to 20 min after bAP stimulation onset for spines with synaptic transmission blocked (red, *n* = 36/14 spines/cells) and control spines (black, *n* = 92/43 spines/cells). (b) The apparent spine size of spines with fluo-5F (blue, *n* = 51/24 spines/cells) and control spines (black, *n* = 89/43 spines/cells) is plotted against the bAP-Ca^2+^ transient 15 to 20 min after bAP stimulation onset.
(EPS)

S5 Fig Scatter plot of enhancement with blockers of intracellular Ca^2+^ release. (a) Averaged baseline amplitude plotted against bAP-Ca^2+^ transient enhancement 15 to 20 min after bAP stimulation onset for spines preincubated with 30 μM CPA (red, *n* = 31/11 spines/cells) and control spines (black, *n* = 92/43 spines/cells). (b) Averaged baseline amplitude plotted against bAP-Ca^2+^ transient enhancement 15 to 20 min after bAP stimulation onset for spines preincubated with 100 μM ryanodine (blue, *n* = 33/17 spines/cells) and control spines (black, *n* = 92/43 spines/cells). (c) Averaged baseline amplitude plotted against bAP-Ca^2+^ transient enhancement 15 to 20 min after bAP stimulation onset for spines preincubated with 10 μM XestosponginC (green, *n* = 68/19 spines/cells) and control spines (black, *n* = 92/43 spines/cells).
(EPS)

S6 Fig Correlation of effect size using different time intervals for measuring the amplitude of bAP-Ca^2+^ transients. bAP-Ca^2+^ transient enhancement measured in a 20 ms time interval starting with the second AP in a doublet plotted against measurement in a 70 ms time interval starting with the first AP (r = 0.85, *n* = 92/43 spines/cells, *p* < 0.0001, Spearman’s rank order test).
(EPS)

S7 Fig Constraint checks for model parameters. (a) Considering only the first two bAPs, the free [Ca^2+^] reaches about 0.5 μM. Then, the RyR opening adds another 0.25 μM. This test was used to tune parameters for the RyR and VGCCs to comply with constraint (b) given in S1 Table. (b) Volume average of bound buffer concentration for 500 μM fluo-5F. From Fig 1C, one can approximate the RyR receptor contribution to the fluorescence signal to be about 10–20%. Assuming a linear relation between ∆G/R and the bound buffer concentration, a constraint on the current and the open time of the RyR arises (S1 Table). In the shown simulation, the signal is increased for about 35%. This is still in good accordance with the experiments considering that for the experiments a 70 ms time average was used as peak value, see “Data analysis and statistics”.(c) Fluorescence decay rates for fluo-4FF and fluo-5F. Simulation data was fitted with a simple exponential decay. The thin dashed line shows an exponential decay with the time scale obtained from measurements (constraint S1 Table).
(EPS)

S1 TableModel parameters.(b, f) Estimated from a representative subset of own measurements. For estimating the number of Ca^2+^ ions per doublet, we applied the calibration method described by Yasuda et al. [23] to a subset of 50 spines from 9 cells. Decay time constants of the fluorescence signal are based on datasets of 45 spines from 9 cells (500 μM fluo-5F) and 45 spines from 19 cells (200 μM fluo-4FF). (c) The number of ions per bAP was estimated in [1] to be *N*_*i*_ ≈ 2000. We choose *N*_*i*_ ≈ 500 to comply with the calibrated measurements (b) and constraints (e) and (d). This does not contradict with [1] since their spine size was significantly larger. The amplitude *A*_0_ is given with *N*_*i*_ as $A_{0}=\frac{N_{i}}{N_{a}wd}$, where *N*_*a*_ is the Avogadro constant and *w* and *d* denote the width and depth of the simulation box, respectively. (e) To satisfy constraints (d) we had to reduce the Ryanodine receptor efflux, which could be achieved by shorter open time or reduced efflux. We did this by tuning the current to a slightly smaller value than given in literature, to achieve a mean open time of 2 ms. Note that higher efflux and shorter open time would not contradict with our conclusions of the nanodomain downstream receptor activation. (g) In [64] the mean open time is given by $\tau_{o}=\frac{1}{k_{close,\max}}=\frac{1}{480s^{-1}}\approx 2$ ms.(DOCX)

## Abbreviations

AMPA: α-amino-3-hydroxy-5-methyl-4-isoxazolepropionic acid
AP: action potential
APV: 2R-amino-5-phosphonovaleric acid
bAP: back-propagating action potential
BDNF: Brain Derived Neurotrophic Factor
CPA: Cyclopiazonic acid
ER: endoplasmic reticulum
GABA_A_: γ-Aminobutyric acid type A
IP3R: inositol-trisphosphate receptor
LTD: long term depression
LTP: long term potentiation
LY 367385: (S)-(+)-α-Amino-4-carboxy-2-methylbe­nzeneacetic acid
MEC: medial entorhinal cortex
mGluRs: metabotropic glutamate receptors
MPEP: 2-Methyl-6-(phenylethynyl)pyridine
NBQX: 2,3-dihydroxy-6-nitro-7-sulfamoyl-benzo[f]quinoxaline-2,3-dione
NMDARs: N-methyl-D-aspartate receptors
RyRs: ryanodine receptors
SERCA: sarcoplasmic/endoplasmic reticulum calcium ATPase
(S)-MCPG: (S)-α-Methyl-4-carboxyphenylglycine
(S/N): signal-to-noise ratio
VGCCs: voltage gated Ca^2+^ channels

## References

[1] Sabatini BL, Oertner TG, Svoboda K. The life cycle of Ca(2+) ions in dendritic spines. Neuron. 2002 1 31;33(3):439–52. 11832230 10.1016/s0896-6273(02)00573-1

[2] Berridge MJ, Lipp P, Bootman MD. The versatility and universality of calcium signalling. Nat Rev Mol Cell Biol. 2000 10;1(1):11–21. 11413485 10.1038/35036035

[3] Harris KM, Kater SB. Dendritic spines: cellular specializations imparting both stability and flexibility to synaptic function. Annu Rev Neurosci. 1994;17:341–71. 8210179 10.1146/annurev.ne.17.030194.002013

[4] Bloodgood BL, Sabatini BL. Ca2+ signaling in dendritic spines. Current Opinion in Neurobiology. 2007 6;17(3):345–51. 17451936 10.1016/j.conb.2007.04.003

[5] Higley MJ, Sabatini BL. Calcium Signaling in Dendrites and Spines: Practical and Functional Considerations. Neuron. 2008 9 25;59(6):902–13. 10.1016/j.neuron.2008.08.020 18817730

[6] Yuste R, Denk W. Dendritic spines as basic functional units of neuronal integration. Nature. 1995 6 22;375(6533):682–4. 7791901 10.1038/375682a0

[7] Finch EA, Augustine GJ. Local calcium signalling by inositol-1,4,5-trisphosphate in Purkinje cell dendrites. Nature. 1998 11;396(6713):753–6. 9874372 10.1038/25541

[8] Takechi H, Eilers J, Konnerth A. A new class of synaptic response involving calcium release in dendritic spines. Nature. 1998 11;396(6713):757–60. 9874373 10.1038/25547

[9] Emptage N, Bliss TV, Fine A. Single synaptic events evoke NMDA receptor–mediated release of calcium from internal stores in hippocampal dendritic spines. Neuron. Elsevier; 1999;22(1):115–24. 10027294 10.1016/s0896-6273(00)80683-2

[10] Kovalchuk Y, Eilers J, Lisman J, Konnerth A. NMDA receptor-mediated subthreshold Ca(2+) signals in spines of hippocampal neurons. J Neurosci. 2000 3 1;20(5):1791–9. 10684880 10.1523/JNEUROSCI.20-05-01791.2000 PMC6772937

[11] Raymond CR. Spatial segregation of neuronal calcium signals encodes different forms of LTP in rat hippocampus. The Journal of Physiology. 2005 9 15;570(1):97–111. 16284072 10.1113/jphysiol.2005.098947 PMC1464297

[12] Holbro N, Grunditz A, Oertner TG. Differential distribution of endoplasmic reticulum controls metabotropic signaling and plasticity at hippocampal synapses. Proceedings of the National Academy of Sciences. 2009 9 1;106(35):15055–60. 10.1073/pnas.0905110106 19706463 PMC2736455

[13] Nakamura T, Barbara JG, Nakamura K, Ross WN. Synergistic release of Ca2+ from IP3-sensitive stores evoked by synaptic activation of mGluRs paired with backpropagating action potentials. Neuron. 1999 11;24(3):727–37. 10595522 10.1016/s0896-6273(00)81125-3

[14] Sala C, Segal M. Dendritic spines: the locus of structural and functional plasticity. Physiological Reviews. 2014 1;94(1):141–88. 10.1152/physrev.00012.2013 24382885

[15] Plotkin JL, Shen W, Rafalovich I, Sebel LE, Day M, Chan CS, et al Regulation of dendritic calcium release in striatal spiny projection neurons. Journal of Neurophysiology. 2013 11 15;110(10):2325–36. 10.1152/jn.00422.2013 23966676 PMC3841873

[16] Garaschuk O, Yaari Y, Konnerth A. Release and sequestration of calcium by ryanodine-sensitive stores in rat hippocampal neurones. The Journal of Physiology. 1997;502(1):13–30. 9234194 10.1111/j.1469-7793.1997.013bl.x PMC1159569

[17] Lu YF, Hawkins RD. Ryanodine receptors contribute to cGMP-induced late-phase LTP and CREB phosphorylation in the hippocampus. Journal of Neurophysiology. 2002 9;88(3):1270–8. 12205148 10.1152/jn.2002.88.3.1270

[18] Manita S, Ross WN. Synaptic Activation and Membrane Potential Changes Modulate the Frequency of Spontaneous Elementary Ca2+ Release Events in the Dendrites of Pyramidal Neurons. J Neurosci. 2009 6 17;29(24):7833–45. 10.1523/JNEUROSCI.0573-09.2009 19535595 PMC2756180

[19] Laver DR, Curtis BA. Response of ryanodine receptor channels to Ca2+ steps produced by rapid solution exchange. Biophys J. 1996 8;71(2):732–41. 8842211 10.1016/S0006-3495(96)79272-X PMC1233529

[20] Sutko JL, Airey JA, Welch W, Ruest L. The pharmacology of ryanodine and related compounds. Pharmacol Rev. 1997 3;49(1):53–98. 9085309

[21] Du GG, Guo X, Khanna VK, MacLennan DH. Ryanodine sensitizes the cardiac Ca(2+) release channel (ryanodine receptor isoform 2) to Ca(2+) activation and dissociates as the channel is closed by Ca(2+) depletion. Proc Natl Acad Sci USA. 2001 11 20;98(24):13625–30. 11698671 10.1073/pnas.241516898 PMC61091

[22] Lorincz A, Rozsa B, Katona G, Vizi ES, Tamas G. Differential distribution of NCX1 contributes to spine-dendrite compartmentalization in CA1 pyramidal cells. Proceedings of the National Academy of Sciences. 2007 1 10;104(3):1033–8. 17215351 10.1073/pnas.0605412104 PMC1783359

[23] Yasuda R, Nimchinsky EA, Scheuss V, Pologruto TA, Oertner TG, Sabatini BL, et al Imaging calcium concentration dynamics in small neuronal compartments. Sci STKE. 2004 2 3;2004(219):pl5. 10.1126/stke.2192004pl5 14872098

[24] Sandler VM, Barbara JG. Calcium-induced calcium release contributes to action potential-evoked calcium transients in hippocampal CA1 pyramidal neurons. J Neurosci. 1999 6 1;19(11):4325–36. 10341236 10.1523/JNEUROSCI.19-11-04325.1999 PMC6782593

[25] McPherson PS, Campbell KP. The ryanodine receptor/Ca2+ release channel. J Biol Chem. 1993 7 5;268(19):13765–8. 8390976

[26] Llano I, González J, Caputo C, Lai FA, Blayney LM, Tan YP, et al Presynaptic calcium stores underlie large-amplitude miniature IPSCs and spontaneous calcium transients. Nat Neurosci. 2000 12;3(12):1256–65. 11100146 10.1038/81781

[27] Ross WN. Understanding calcium waves and sparks in central neurons. Nat Rev Neurosci. 2012 2 8;13(3):157–68. 10.1038/nrn3168 22314443 PMC4501263

[28] Ozaki H, Hori M, Kim Y-S, Kwon S-C, Ahn D-S, Nakazawa H, et al Inhibitory mechanism of xestospongin-C on contraction and ion channels in the intestinal smooth muscle. British Journal of Pharmacology. 2002 12;137(8):1207–12. 12466229 10.1038/sj.bjp.0704988 PMC1573613

[29] Yasuda R, Sabatini BL, Svoboda K. Plasticity of calcium channels in dendritic spines. Nat Neurosci. 2003 8 24;6(9):948–55. 12937422 10.1038/nn1112

[30] Hoogland TM, Saggau P. Facilitation of L-Type Ca2+ Channels in Dendritic Spines by Activation of 2 Adrenergic Receptors. J Neurosci. 2004 9 29;24(39):8416–27. 15456814 10.1523/JNEUROSCI.1677-04.2004 PMC6729902

[31] Sabatini BL, Svoboda K. Analysis of calcium channels in single spines using optical fluctuation analysis. Nature. 2000 11 30;408(6812):589–93. 11117746 10.1038/35046076

[32] Chalifoux JR, Carter AG. GABAB receptor modulation of voltage-sensitive calcium channels in spines and dendrites. J Neurosci. 2011 3 16;31(11):4221–32. 10.1523/JNEUROSCI.4561-10.2011 21411663 PMC3061967

[33] Kim J, Jung S-C, Clemens AM, Petralia RS, Hoffman DA. Regulation of Dendritic Excitability by Activity-Dependent Trafficking of the A-Type K+ Channel Subunit Kv4.2 in Hippocampal Neurons. Neuron. 2007 6 21;54(6):933–47. 17582333 10.1016/j.neuron.2007.05.026 PMC1950443

[34] Ngo-Anh TJ, Bloodgood BL, Lin M, Sabatini BL, Maylie J, Adelman JP. SK channels and NMDA receptors form a Ca2+-mediated feedback loop in dendritic spines. Nat Neurosci. 2005 5;8(5):642–9. 15852011 10.1038/nn1449

[35] Bloodgood BL, Sabatini BL. Neuronal activity regulates diffusion across the neck of dendritic spines. Science. 2005 11 4;310(5749):866–9. 16272125 10.1126/science.1114816

[36] Grunditz A, Holbro N, Tian L, Zuo Y, Oertner TG. Spine Neck Plasticity Controls Postsynaptic Calcium Signals through Electrical Compartmentalization. J Neurosci. 2008 12 10;28(50):13457–66. 10.1523/JNEUROSCI.2702-08.2008 19074019 PMC6671740

[37] Bloodgood BL, Giessel AJ, Sabatini BL. Biphasic synaptic Ca influx arising from compartmentalized electrical signals in dendritic spines. PLoS Biol. 2009 9;7(9):e1000190 10.1371/journal.pbio.1000190 19753104 PMC2734993

[38] Harnett MT, Makara JK, Spruston N, Kath WL, Magee JC. Synaptic amplification by dendritic spines enhances input cooperativity. Nature. 2012 11 22;491(7425):599–602. 10.1038/nature11554 23103868 PMC3504647

[39] Gulledge AT, Carnevale NT, Stuart GJ. Electrical Advantages of Dendritic Spines. Mansvelder HD, editor. PLoS ONE. 2012 4 20;7(4):e36007 10.1371/journal.pone.0036007 22532875 PMC3332048

[40] Yuste R. Electrical Compartmentalization in Dendritic Spines. Annu Rev Neurosci. 2013 7 8;36(1):429–49. 23724997 10.1146/annurev-neuro-062111-150455

[41] Nägerl UV, Eberhorn N, Cambridge SB, Bonhoeffer T. Bidirectional Activity-Dependent Morphological Plasticity in Hippocampal Neurons. Neuron. 2004 12;44(5):759–67. 15572108 10.1016/j.neuron.2004.11.016

[42] Holthoff K, Zecevic D, Konnerth A. Rapid time course of action potentials in spines and remote dendrites of mouse visual cortex neurons. The Journal of Physiology. 2010 4 1;588(Pt 7):1085–96. 10.1113/jphysiol.2009.184960 20156851 PMC2852997

[43] Palmer LM, Stuart GJ. Membrane potential changes in dendritic spines during action potentials and synaptic input. J Neurosci. 2009 5 27;29(21):6897–903. 10.1523/JNEUROSCI.5847-08.2009 19474316 PMC6665597

[44] Popovic MA, Gao X, Carnevale NT, Zecevic D. Cortical Dendritic Spine Heads Are Not Electrically Isolated by the Spine Neck from Membrane Potential Signals in Parent Dendrites. Cerebral Cortex. 2014 2;24(2):385–95. 10.1093/cercor/bhs320 23054810 PMC3888368

[45] Araya R, Jiang J, Eisenthal KB, Yuste R. The spine neck filters membrane potentials. Proc Natl Acad Sci USA. 2006 11 21;103(47):17961–6. 17093040 10.1073/pnas.0608755103 PMC1693855

[46] Waters J, Schaefer A, Sakmann B. Backpropagating action potentials in neurones: measurement, mechanisms and potential functions. Prog Biophys Mol Biol. 2005 1;87(1):145–70. 15471594 10.1016/j.pbiomolbio.2004.06.009

[47] Kato HK, Watabe AM, Manabe T. Non-Hebbian Synaptic Plasticity Induced by Repetitive Postsynaptic Action Potentials. J Neurosci. 2009 9 9;29(36):11153–60. 10.1523/JNEUROSCI.5881-08.2009 19741122 PMC6665946

[48] Bukalo O, Campanac E, Hoffman DA, Fields RD. Synaptic plasticity by antidromic firing during hippocampal network oscillations. Proceedings of the National Academy of Sciences. 2013 3 26;110(13):5175–80. 10.1073/pnas.1210735110 23479613 PMC3612622

[49] Dudek SM, Fields RD. Somatic action potentials are sufficient for late-phase LTP-related cell signaling. Proceedings of the National Academy of Sciences. 2002 3 12;99(6):3962–7. 11891337 10.1073/pnas.062510599 PMC122631

[50] Hayama T, Noguchi J, Watanabe S, Takahashi N, Hayashi-Takagi A, Ellis-Davies GCR, et al GABA promotes the competitive selection of dendritic spines by controlling local Ca2+ signaling. Nat Neurosci. 2013 10;16(10):1409–16. 10.1038/nn.3496 23974706 PMC4135703

[51] Zalk R, Lehnart SE, Marks AR. Modulation of the Ryanodine Receptor and Intracellular Calcium. Annu Rev Biochem. 2007 6 7;76(1):367–85. 17506640 10.1146/annurev.biochem.76.053105.094237

[52] Zhao W, Meiri N, Xu H, Cavallaro S, Quattrone A, Zhang L, et al Spatial learning induced changes in expression of the ryanodine type II receptor in the rat hippocampus. FASEB J. 2000 2;14(2):290–300. 10657985 10.1096/fasebj.14.2.290

[53] Adasme T, Haeger P, Paula-Lima AC, Espinoza I, Mercedes Casas-Alarcon M, Angelica Carrasco M, et al Involvement of ryanodine receptors in neurotrophin-induced hippocampal synaptic plasticity and spatial memory formation. Proc Natl Acad Sci USA. 2011;108(7):3029–34. 10.1073/pnas.1013580108 21282625 PMC3041089

[54] Li Q, Rothkegel M, Xiao ZC, Abraham WC, Korte M, Sajikumar S. Making Synapses Strong: Metaplasticity Prolongs Associativity of Long-Term Memory by Switching Synaptic Tag Mechanisms. Cerebral Cortex. 2014 1 10;24(2):353–63. 10.1093/cercor/bhs315 23048020

[55] Liu X, Betzenhauser MJ, Reiken S, Meli AC, Xie W, Chen B-X, et al Role of Leaky Neuronal Ryanodine Receptors in Stress- Induced Cognitive Dysfunction. Cell. 2012 8 31;150(5):1055–67. 10.1016/j.cell.2012.06.052 22939628 PMC3690518

[56] Goussakov I, Miller MB, Stutzmann GE. NMDA-Mediated Ca2+ Influx Drives Aberrant Ryanodine Receptor Activation in Dendrites of Young Alzheimer's Disease Mice. J Neurosci. 2010 9 8;30(36):12128–37. 10.1523/JNEUROSCI.2474-10.2010 20826675 PMC2944253

[57] Stranahan AM, Mattson MP. Selective Vulnerability of Neurons in Layer II of the Entorhinal Cortex during Aging and Alzheimer's Disease. Neural Plasticity. 2010;2010: 108190 10.1155/2010/108190 21331296 PMC3039218

[58] Beed P, Bendels MHK, Wiegand HF, Leibold C, Johenning FW, Schmitz D. Analysis of excitatory microcircuitry in the medial entorhinal cortex reveals cell-type-specific differences. Neuron. 2010 12 22;68(6):1059–66. 10.1016/j.neuron.2010.12.009 21172609

[59] Holtmaat AJGD, Trachtenberg JT, Wilbrecht L, Shepherd GM, Zhang X, Knott GW, et al Transient and Persistent Dendritic Spines in the Neocortex In Vivo. Neuron. 2005 1;45(2):279–91. 15664179 10.1016/j.neuron.2005.01.003

[60] Blatt M, Dedner A, Engwer C, Klöfkorn R, Ohlberger M. A generic grid interface for parallel and adaptive scientific computing. Part I: abstract framework. Computing. 2008;82:103–119.

[61] Bastian P, Blatt M, Dedner A, Engwer C, Klöfkorn R. A generic grid interface for parallel and adaptive scientific computing. Part II: Implementation and tests in DUNE. Computing. 2008;82:121–138.

[62] Rückl M, Parker I, Marchant JS, Nagaiah C, Johenning FW, Rüdiger S. Modulation of Elementary Calcium Release Mediates a Transition from Puffs to Waves in an IP3R Cluster Model. Gabhann FM, editor. PLoS Comp Biol. 2015 1 8;11(1):e1003965 10.1371/journal.pcbi.1003965 25569772 PMC4288706

[63] Sobie EA, Dilly KW, Santos Cruz dos J, Lederer WJ, Jafri MS. Termination of cardiac Ca(2+) sparks: an investigative mathematical model of calcium-induced calcium release. Biophys J. 2002 7;83(1):59–78. 12080100 10.1016/s0006-3495(02)75149-7 PMC1302127

[64] Ramay HR, Liu OZ, Sobie EA. Recovery of cardiac calcium release is controlled by sarcoplasmic reticulum refilling and ryanodine receptor sensitivity. Cardiovasc Res. 2011 9 1;91(4):598–605. 10.1093/cvr/cvr143 21613275 PMC3156908

[65] Bezprozvanny I, Watras J, Ehrlich BE. Bell-shaped calcium-response curves of Ins(1,4,5)P3- and calcium-gated channels from endoplasmic reticulum of cerebellum. Nature. 1991 6 27;351(6329):751–4. 1648178 10.1038/351751a0

