# Supplementary material for: Ryanodine Receptor Activation Induces Long-Term Plasticity of Spine Calcium Dynamics
Source: PLoS Biol. 2015 Jun 22;13(6):e1002181. doi: 10.1371/journal.pbio.1002181 (PMC4476683; doi:10.1371/journal.pbio.1002181)
Supplement: S1 Table — (b, f) Estimated from a representative subset of own measurements. For estimating the number of Ca2+ ions per doublet, we applied the calibration method described by Yasuda et al. [23] to a subset of 50 spines from 9 cells. Decay time constants of the fluorescence signal are based on datasets of 45 spines from 9 cells (500 μM fluo-5F) and 45 spines from 19 cells (200 μM fluo-4FF). (c) The number of ions per bAP was estimated in [1] to be N i ≈ 2000. We choose N i ≈ 500 to comply with the calibrated measurements (b) and constraints (e) and (d). This does not contradict with [1] since their spine size was significantly larger. The amplitude A 0 is given with N i as A0=NiNawd, where N a is the Avogadro constant and w and d denote the width and depth of the simulation box, respectively. (e) To satisfy constraints (d) we had to reduce the Ryanodine receptor efflux, which could be achieved by shorter open time or reduced efflux. We did this by tuning the current to a slightly smaller value than given in literature, to achieve a mean open time of 2 ms. Note that higher efflux and shorter open time would not contradict with our conclusions of the nanodomain downstream receptor activation. (g) In [64] the mean open time is given by τo=1kclose,max=1480s−1≈2 ms. (DOCX) [file pbio.1002181.s009.docx]

Constraints for model parameters

|  | Parameter | Constrained by | published/measured | used | Reference |
| --- | --- | --- | --- | --- | --- |
| (a) | $B_{e}$, $k_{e}^{+}$,  $k_{e}^{-}$, $D_{e}$ | Endogenous buffer capacity | $\kappa=24\pm11$ | $\kappa\approx19$ | [1] |
| (b) | $A_{0}$ | VGCC contribution to Ca²⁺ peak amplitudes | $0.4-0.6$µM | $0.4-0.75$  µM |  |
| (c) | $A_{0}$ | Number of Ca²⁺ ions per bAP | $2000$ | $500$ | [1] |
| (d) | $P_{RyR}$, $E_{0}$ | Contribution of RyR influx to fluorescence peak amplitude | $10-20 \%$ | $35 \%$ | Fig. S7b  Fig. 1c |
| (e) | $P_{RyR}$, $E_{0}, R_{c}$ | Channel current amplitude | $0.5-1.5$pA | $\sim0.1$pA | [2] |
| (f) | $\lambda, K_{SERCA},$  $P_{SERCA}$ | Measurements of fluorescence decay rate | $60$ms (fluo-4FF)  $250$ms (fluo-5F) | $43$ms  $225$ms | Fig. S7c |
| (g) | $\tau_{o}$ | Ryanodine open time | 2 ms | 2 ms | [3] |

Reaction-diffusion parameters

| Description | Symbol | Value | Unit | Reference or  constraint |
| --- | --- | --- | --- | --- |
| [Ca^2+^] diffusion coefficient | $D_{c}$ | 223 | µm²/s | [4] |
| [Ca^2+^] resting concentration | $c_{0}$ | 50 | nM | $70\pm30$nM [1] |
| luminal resting concentration | $E_{0}$ | 700 | µM | (d) and [5] |
| SERCA flux coefficient | $P_{SERCA}$ | 20000 | nm µM/s | (f) |
| SERCA dissociation coefficient | $K_{SERCA}$ | 0.2 | µM | [6], (f) |
| extrusion coefficient | $\lambda$ | 750 | 1/s | (f) |
| RyR channel radius | $R_{c}$ | 6 | nm | (e) |
| RyR channel flux coefficient | $P_{RyR}$ | $6.54\times{10}^{6}$ | nm/s | (e) |
| Domain size | $w\times d\times h$ | $300\times300\times200$ | nm^3^ | Fig. S2d |
| RyR channel open time | $\tau_{o}$ | 2 | ms | [7], (i) |

Endogenous buffer parameters

| total concentration | $B_{e}$ | 200 | µM | [1] and (a) |
| --- | --- | --- | --- | --- |
| on rate | $k_{e}^{+}$ | 30 | 1/(µM s) | [1] and (a) |
| off rate | $k_{e}^{-}$ | 300 | 1/s | [1] and (a) |
| diffusion coefficient | $D_{e}$ | 0.1 | µm²/s | assumption |

**F**luo-4FF buffer parameters

| total concentration | $B_{d}$ | 200 | µM | Experimental setup |
| --- | --- | --- | --- | --- |
| on rate | $k_{d}^{+}$ | 30 | 1/(µM s) | [6] |
| off rate | $k_{d}^{-}$ | 300 | 1/s | [6] |
| diffusion coefficient | $D_{d}$ | 20 | µm²/s | assumption |

Fluo-5F buffer parameters

| total concentration | $B_{d}$ | 200 or 500 | µM | Experimental setup |
| --- | --- | --- | --- | --- |
| on rate | $k_{d}^{+}$ | 150 | 1/(µM s) | [6] |
| off rate | $k_{d}^{-}$ | 300 | 1/s | [6] |
| diffusion coefficient | $D_{d}$ | 20 | µm²/s | assumption |

VGCC parameters

| pulse width | $\sigma$ | 1 | ms | assumption |
| --- | --- | --- | --- | --- |
| ions per pulse | $N_{i}$ | 500 |  | (b) |
| influx amplitude | $A_{0}$ | 9225 | nm µM | (c) |
| pulse delay | $\tau$ | 10 | ms | Experimental setup |
| # of pulses | $N$ | 2 or 5 |  | Experimental setup |

Ryanodine receptor model parameters

| Dissociation coefficient | $K_{d}$ | 1.06 | µM | [8] |
| --- | --- | --- | --- | --- |
| RyR luminal dependence factor | $\alpha$ | 0.15 |  | Chosen to achieve $K_{d}\approx1$µM |
| RyR luminal dependence factor | $K_{m}$ | 720 | µM | Chosen to achieve $K_{d}\approx1$µM |
| Maximum RyR open rate | $\rho_{+}$ | 30.000 | 1/s | [7] |
| RyR close rate | $\rho_{-}$ | 480 | 1/s | [7] |

1. Sabatini BL, Oertner TG, Svoboda K. The life cycle of Ca(2+) ions in dendritic spines. Neuron. 2002 Jan 31;33(3):439–52.

2. Fill M, Copello JA. Ryanodine Receptor Calcium Release Channels. Physiol Rev. 2002 Oct;82(4):893-922.

3. Ramay HR, Liu OZ, Sobie EA. Recovery of cardiac calcium release is controlled by sarcoplasmic reticulum refilling and ryanodine receptor sensitivity. Cardiovasc Res. 2011 Sep 1;91(4):598–605.

4. Allbritton N, Meyer T and Stryer L. Range of messenger action of Ca2+ ion and

IP3. Science. 1992 Dec 11;258(5089):1812-5.

5. Ullah G, Parker I, Mak DO, Pearson JE. Multi-scale data-driven modeling and

observation of calcium puffs. Cell Calcium. 2012 Aug;52(2):152-60.

6. Yasuda R, Nimchinsky EA, Scheuss V, Pologruto TA, Oertner TG, Sabatini BL, et al. Imaging calcium concentration dynamics in small neuronal compartments. Sci STKE. 2004 Feb 3;2004(219):pl5.

7. Ramay HR, Liu OZ, Sobie EA. Recovery of cardiac calcium release is controlled by sarcoplasmic reticulum refilling and ryanodine receptor sensitivity. Cardiovasc Res. 2011 Sep 1;91(4):598–605.

8. Bezprozvanny I, Watras J, Ehrlich BE. Bell-shaped calcium-response curves of Ins(1,4,5)P3- and calcium-gated channels from endoplasmic reticulum of cerebellum. Nature. 1991 Jun 27;351(6329):751–4.
